# Psoralen mapping reveals a bacterial genome supercoiling landscape dominated by transcription

**DOI:** 10.1093/nar/gkac244

**Published:** 2022-04-14

**Authors:** Bryan J Visser, Sonum Sharma, Po J Chen, Anna B McMullin, Maia L Bates, David Bates

**Affiliations:** Graduate Program in Integrative Molecular and Biomedical Sciences, Baylor College of Medicine, Houston, TX 77030, USA; Molecular and Human Genetics, Baylor College of Medicine, Houston, TX 77030, USA; Molecular Virology and Microbiology, Baylor College of Medicine, Houston, TX 77030, USA; Molecular Virology and Microbiology, Baylor College of Medicine, Houston, TX 77030, USA; Molecular and Human Genetics, Baylor College of Medicine, Houston, TX 77030, USA; Graduate Program in Integrative Molecular and Biomedical Sciences, Baylor College of Medicine, Houston, TX 77030, USA; Molecular and Human Genetics, Baylor College of Medicine, Houston, TX 77030, USA; Molecular Virology and Microbiology, Baylor College of Medicine, Houston, TX 77030, USA; Dan L Duncan Comprehensive Cancer Center, Baylor College of Medicine, Houston, TX 77030, USA

## Abstract

DNA supercoiling is a key regulator of all DNA metabolic processes including replication, transcription, and recombination, yet a reliable genomic assay for supercoiling is lacking. Here, we present a robust and flexible method (Psora-seq) to measure whole-genome supercoiling at high resolution. Using this tool in *Escherichia coli*, we observe a supercoiling landscape that is well correlated to transcription. Supercoiling twin-domains generated by RNA polymerase complexes span 25 kb in each direction – an order of magnitude farther than previous measurements in any organism. Thus, ribosomal and many other highly expressed genes strongly affect the topology of about 40 neighboring genes each, creating highly integrated gene circuits. Genomic patterns of supercoiling revealed by Psora-seq could be aptly predicted from modeling based on gene expression levels alone, indicating that transcription is the major determinant of chromosome supercoiling. Large-scale supercoiling patterns were highly symmetrical between left and right chromosome arms (replichores), indicating that DNA replication also strongly influences supercoiling. Skew in the axis of symmetry from the natural *ori-ter* axis supports previous indications that the rightward replication fork is delayed several minutes after initiation. Implications of supercoiling on DNA replication and chromosome domain structure are discussed.

## INTRODUCTION

Cellular DNA exists in a wide range of topological states in which the double helix is over or under twisted from its relaxed contour of ∼10.5 bp per helical turn. This upset in twist imparts torsional strain on the DNA duplex that causes it to wrap around itself, a phenomenon known as supercoiling. Supercoiling is a fundamental and necessary characteristic of chromosomes in all cells, with major roles in DNA compaction and strand opening operations. By reducing or increasing the free energy requirement of duplex melting at transcription start sites, supercoiling strongly modulates gene expression, particularly in bacteria, in which initial loading of RNA polymerase (RNAP) is not aided by ATP hydrolysis (1). Similarly, open complex formation at origins of replication are dependent and partly regulated by supercoiling (2). DNA supercoiling also influences the stability and migration of three-way and four-way junctions, and thus affects replication fork progression (3), R-loop formation (4) and genetic recombination (5). Most visibly, supercoiling modulates DNA compaction and three-dimensional organization, affecting chromosome packaging, segregation and even gene expression – by promoting interactions between genes and distantly located regulatory elements via rapid branching and diffusion of plectonemic loops (6).

Much supercoiling is generated by DNA translocases (e.g. DNA and RNA polymerase), which, according to Liu and Wang's (7) twin-domain model, act as moving topological barriers generating a region of over-twist (positive supercoiling) ahead of the complex, and a region of under-twist (negative supercoiling) behind it. A single DNA replication complex (replisome) is expected to create particularly high levels of supercoiling (3), as they frequently migrate 100′s of kb at a time without stopping (8). We previously showed that newly replicated DNA duplexes in *E. coli* are wrapped around each other forming long precatenanes (9). Such structures are thought to result from accumulated positive supercoiling in front of the replisome that forces the fork to rotate in the opposite direction, entwining sister chromatids. Although *E. coli*, like all bacteria, have two abundant topoisomerases that reduce positive supercoiling, DNA gyrase and Topo IV (10), the presence of precatenanes at replication forks suggests that these enzymes are frequently outpaced by replication-induced supercoiling, which can reach 100 helical twists per second per fork (3). The resulting wave of positive supercoiling through the genome has been shown to disrupt gene expression (11) possibly coordinating expression of genes regulating the cell cycle to the timing of DNA replication. The net supercoiling effect of transcribing RNA polymerase (RNAP) complexes, which number about 10,000 in rapidly growing *E. coli* cells (12), likely exceeds that of replisomes. Transcription-coupled DNA supercoiling (TCDS) is known to have a profound impact on the expression of nearby genes (13,14), and supercoiling-sensitive genes may be organized into networks, controlling spatial dynamics (15) and permitting auto-regulation of related genes (16,17).

Once generated, diffusion of supercoiling through the genome is regulated by three main mechanisms: selective removal of supercoils by topoisomerases, transitory ‘constraint’ of supercoils by DNA-binding proteins, and limitation of where supercoils can diffuse by topological domain barriers. With the exception of bacterial DNA gyrase (and reverse gyrase in thermophilic archaea), all known topoisomerases function as ‘relaxases’, reducing supercoiled DNA towards the relaxed state (18). Cells maintain a slightly negative supercoiled state presumably to enable strand opening reactions. The typically supercoiled *E. coli* chromosome has a supercoiling density of σ = −0.06, or ∼6 fewer helical turns per 100 than would be present if it were relaxed. This negative state is thought to be maintained by selective relaxation of positive supercoils at replication and transcription twin-domains (19–21). In bacteria, DNA gyrase can introduce negative supercoils ‘uphill’ (within DNA tracts that are already negatively supercoiled), utilizing ATP hydrolysis to catalyze a unidirectional double strand cleavage/passage reaction (22). A significant fraction of supercoiling is also constrained by DNA binding proteins, namely nucleosomes (23) and bacterial histone-like proteins (eg. 24). These proteins can direct the creation of new supercoils, despite not having the ability to carry out strand cleavage, by contorting DNA and introducing writhe. Writhed segments are actively relaxed by topoisomerases (25), thus introducing supercoils. These supercoils are said to be constrained because they are locked in place by the bound nucleoid protein. Constrained supercoils are converted into freely diffusible supercoils upon release of the protein. It is estimated that about half of all supercoils in eukaryotes and bacteria are constrained by proteins (26); however, the degree to which constrained supercoiling is utilized as a genetic regulatory mechanism is unclear (27,28).

Supercoils can also be prevented from interacting with genetic elements by topological barriers, which block the passage of diffusible supercoils, thus defining topological domains. Barriers are formed by DNA binding proteins that link DNA segments to cell structure or another DNA segment, and by transcription complexes, especially at highly transcribed genes (26). Knowledge of the topological domain structure of chromosomes is very limited due to technical challenges in measuring supercoiling (below). Best estimates of topological barriers in *E. coli* range from one barrier every 10 kb (29) to one barrier every 100 kb (30), based on the effects of double strand breaks on the expression of relaxation-inhibited genes (29) or the binding of the supercoiling-sensitive DNA intercalator, psoralen (30). An estimate in the closely related *Salmonella* Typhimurium based on disruption of supercoiling-dependent recombination between two γδ transposons suggests that topological barriers are spaced about 25 kb apart (31). These studies also suggest that barriers are stochastically positioned throughout the genome in bacteria (29,31). The *E. coli* chromosome is further organized into six ∼800 kb macrodomains exhibiting independent recombination and spatial dynamics (32). Macrodomains coincide closely with chromosome interaction domains (CIDs) identified by 3C experiments (33). These domains are typically bounded by ribosomal or other highly expressed genes, suggesting that the sites of highest transcription may act as topological ‘mega-barriers’. Analogous topologically associating domains (TADs) identified in eukaryotes by 3C are also flanked by highly expressed genes (34). TADs (and perhaps CIDs) may form by TCDS-mediated loop extrusion, which would imply that they are independently supercoiled (35). However, preliminary examinations of supercoiling in bacterial CIDs (6) and mammalian TADs (23) do not indicate independent supercoiling, so the relationship between supercoiling and large 3C-identified domains is still unclear.

DNA supercoiling freely converts between many structural forms and is highly sensitive to changes in ionic concentration, temperature, and molecular crowding. This ethereal nature of supercoiling makes it very difficult to quantify experimentally, especially in vivo, and existing assays are technically challenging and have poor sensitivity. Most genomic supercoiling assays utilize the DNA intercalator psoralen, which binds duplex DNA at a rate directly proportional to helical tension (twist), with binding being most strong to underwound regions (36). Psoralen binding to purified DNA has a wide dynamic range, increasing about 1.2-fold (120%) between relaxed and moderately negatively supercoiled (σ = −0.07) molecules in vitro (36,37). However, a much narrower range of psoralen binding (about 0.2-fold or 20%) is typically seen along chromosomes in vivo (28,38), which typically quantify binding by chromatin immunoprecipitation of psoralen-bound fragments on genomic DNA arrays (ChIP-chip). Decreased sensitivity is likely due to several factors including inefficient UV-mediated crosslinking and purification of psoralen-bound fragments, as well as limited resolution and binding response of DNA arrays.

To address these issues, we have developed a highly sensitive psoralen-based supercoiling assay (Psora-seq) that is fast, reliable, and uses commercially available materials. Using this method, we show that transcription from highly expressed ribosomal genes disrupts supercoiling over a 50 kb region—substantially farther than previously measured. Models of genome supercoiling, generated by extrapolating the supercoiling dynamics observed at ribosomal genes to all other genes, showed good agreement with the supercoiling profile from Psora-seq, suggesting that transcription is a major determinant of supercoiling in the *E. coli* genome. Our method also revealed an unexpected bilateral symmetric pattern of supercoiling over the chromosome that is centered on a skewed origin—terminus replication axis. The interplay between transcription and replication and their effects on genome supercoiling are discussed.

## MATERIALS AND METHODS

### Bacterial strains and growth conditions

The wild-type *E. coli* strain MG1655 was used for all experiments except for the *invA* inversion strain, which has a 18.4 kb kanamycin marked inversion including the *rrnA* operon (39) introduced into MG1655 by P1 transduction. All cells were grown in LB medium at 37°C from a 1:2000 dilution to mid-exponential phase at O.D.600 = 0.2. Late-exponential phase and stationary phase samples were grown to OD_600_ = 2 and 7, respectively. To relax cellular DNA supercoiling, the DNA nicking antibiotic bleomycin (ThermoFisher cat no. J60727) was added to 70 μg/ml, which resulted in DNA fragment sizes ∼0.1–1 kb (Supplementary Figure S4E, 40). Bleomycin was added at OD 0.2 and cells were further incubated for 10 minutes prior to psoralen addition. To inhibit RNAP, OD 0.2 cells were treated with rifampicin (Sigma-Aldrich cat no. R7382) at 150 μg/ml for one hour prior to psoralen addition. To inhibit type-II topoisomerases, norfloxacin (Sigma-Aldrich cat no. N9890) was added to 10 μg/ml at OD 0.2 and cells were incubated for 20 or 60 min before psoralen addition as indicated. The shorter incubation time for norfloxacin was chosen to detect supercoiling changes at ribosomal twin-domains prior to transcription arrest, which occurred by 60 min of incubation (Supplementary Figure S9E, F).

### Psoralen crosslinking and affinity purification

The Psora-seq method was developed from prior psoralen studies (23,28,36–38,41), optimized by testing different conditions of psoralen concentration, incubation temperature and time, and crosslinking energy. Those conditions resulting in the highest DNA yield per ml cells after purification with streptavidin beads were selected (data not shown). The resulting Psora-seq protocol, used for all experiments described here, is as follows. 100 ml of exponentially growing cells were chilled for 3 minutes in ice water, then pelleted by centrifugation at 4000 × g for 5 min and resuspended in 10 ml cold TE with 2.5 μg/ml biotinylated psoralen (EZ-Link Psoralen-PEG3-Biotin, ThermoFisher). For late-exponential and stationary phase samples, culture volumes were reduced to 10 and 2 ml, respectively. Cells were incubated on ice for 10 minutes in the dark, then crosslinked in an open 10 cm polystyrene petri dish with 2.4 J/cm^2^ (total energy) 365 nm UV light in a UVP CL-3000L crosslinker. Genomic DNA was prepared by the CTAB method (42), and resuspended in 400 μl of TE buffer. DNA was sheared to an average size of about 500 bp by sonication in ice water using a cup sonicator (Branson model 250) for 6 cycles at output level 4 (40 s on, 20 s off at 50% duty cycle).

To prepare for affinity purification, 100 μl of 1 μm diameter streptavidin-coupled magnetic beads (Invitrogen Dynabeads MyOne Streptavidin T1) were washed according to the manufacturer's instructions, then half the DNA (200 μl) is added to the beads and rotated for 1 hour at room temperature in the dark, followed by 16 h rotation at 4°C in the dark. Unbound DNA was removed by washing beads according to the manufacturer's instructions, then resuspended in 100 μl 95% deionized formamide (10 mM EDTA, pH 8) and heated to 95°C for 5 min to release bound DNA. The DNA/bead mixture was pipetted repeatedly for 30 s, then placed on a magnetic stand for 30 s before collecting the eluate. The remaining 200 μl of sheared DNA (input sample) was prepared by heating in formamide as above. Affinity purified and input samples were precipitated and resuspended in 10 μl TE each. DNA was quantified by Qubit Fluorometer (ThermoFisher) and diluted to 0.2 ng/μl in TE for sequencing.

### Sequencing

Sequencing libraries were created using the Nextera XT DNA Sample Preparation Kit according to manufacturer protocol except that sequencing libraries were manually normalized by quantifying by qPCR, adjusting each library to a final concentration of 2 nM prior to pooling. PCR amplification was performed on a 7900HT Real-Time PCR System (Applied BioSystems) using the KAPA SYBR FAST qPCR kit and primers specific to the adapter sequences on either side of the *E. coli* fragment (forward primer 5′-AATGATACGGCGACCACCGAGAT-3′, reverse primer 5′-CAAGCAGAAGACGGCATACGA-3′). A standard curve was generated using KAPA DNA Standards for Illumina platforms (cat# 07960409001) and library fragment size was estimated by agarose gel electrophoresis. Pooled libraries were adjusted to a final concentration of 6-20 pM before denaturing and sequencing. Paired-end sequencing was performed on either an Illumina MiSeq sequencer, or an Illumina NextSeq sequencer, with a 150-cycle MiSeq V3 reagent kit (Illumina) using the re-sequencing workflow. Sequencing reads were aligned to an MG1655 reference genome (accession NC_000913) using Illumina integrated BaseSpace sequencing data analysis software. Aligned reads (0.5–3 million per sample) were extracted, sorted into 1-kb bins, and exported to Excel format using two custom Matlab scripts, sequencingcompile.m and sequencingcompile2.m (https://github.com/DavidBatesLab/Matlab-scripts.git). The 35 bins containing ribosomal genes, which cannot be accurately mapped to the genome due to high repetitive sequence, were replaced with average values from the surrounding 6 kb and relative reads per bin were normalized to genome average. Psora-seq enrichment was calculated by dividing normalized pulldown values by normalized input values then log_2_ transformed.

### Analysis of supercoiling at ribosomal operons

To measure supercoiling produced by ribosomal gene transcription, Psora-seq data from 80 kb surrounding each of the seven *E. coli* ribosomal operons was co-oriented by the direction of *rrn* transcription and aligned to the midpoint of each operon. To quantify the relative change in supercoiling generated by an operon, psoralen profiles were normalized so that the mean log_2_ binding value over the 80-kb range was zero. Aligned and normalized data from all 7 operons and from 6 independent samples were then averaged together to create a consensus ribosomal operon psoralen profile (Figure 4B). Twin-domain amplitude, the maximal change in psoralen binding across the operon midpoint, and magnitude, the farthest distance that a change in psoralen binding could be detected, were estimated by fitting linear regression lines on either side of the operon. Regressions included the first 10 kb from the operon midpoint, then extended one kb at a time until the regression line reached its maximal Pearson correlation coefficient. Amplitude and magnitude of the twin-domains is given by the *y* and *x* intercepts, respectively. Statistical ranges were determined from the intercepts of the 95% confidence intervals.

### Modeling genome supercoiling from transcription

To model genome supercoiling from transcriptome data, we estimated the supercoiling twin-domain of all 2,598 transcription units (837 operons and 1761 single genes), then summed these predicted twin-domains to create a composite twin-domain map. Individual twin-domains were patterned after ribosomal operon twin-domains scaled by the relative rate of transcription. Transcription rates were obtained from two data sets, RNAP binding by ChIP-chip (43) and mRNA frequency quantified by RNA-seq (44). For every operon, twin-domain amplitude (height at the operon midpoint) was calculated as the average ribosomal operon amplitude (±0.38) multiplied by its relative rate of transcription (the ratio of each operon's reported expression level to that of the average ribosomal operon). Twin-domain magnitude (distance away from the operon experiencing a change in psoralen binding) was assumed to be the same for all genes and was set to the magnitude determined for ribosomal operons: 23 and 25 kb for negative and positive domains, respectively. The resulting composite modeled genome supercoiling profile was normalized to the minimum and maximum psoralen binding values from the Psora-seq profile to facilitate comparisons.

### Processing of binding data for nucleoid proteins and topoisomerases

Genomic protein binding information was obtained from previous ChIP-seq studies (27,45,46). Mid-exponential (ME) data from (27) were selected for H-NS, FIS and RpoB (RNAP). For HU (45), data from both subunits, HupA and HupB, were averaged. For Topo I binding, two replicates of TopA-FLAG ChIP-seq data from the Cai lab (GEO sample GSM1696179) were normalized to remove replication-dependent copy number effects, then averaged. For Topo IV binding (46), two replicates of ParE and one replicate of ParC were averaged. Topo IV cleavage data from the same study was created by sequencing DNA from norfloxacin-induced cleavage complexes (46). DNA gyrase binding data was acquired from a previous ChIP-chip study (47), averaging data from 4 replicates. Gyrase cleavage information was acquired from (48), averaging ChIP-seq data from ciprofloxacin, microcin B17, and oxolinic acid. Raw reads from all ChIP-seq data were aligned to the MG1655 reference genome, sorted into 1-kb bins spanning the chromosome, normalized by calculating the number of reads relative to chromosome average, then the immunoprecipitated sample was divided by the input sample (IP/input), log_2_ transformed, and converted to standard (*z*) score. For ChIP-chip data, microarray coordinates were converted to MG1655 NC_000913.3, then sorted into 1-kb bins and converted to z-scores as above. Psora-seq data (log_2_ reads IP/input) were also converted to z-scores.

### Statistics

Graphing and statistical analyses were performed in Microsoft Excel with the Real Statistics Resource Pack for Macintosh (www.real-statistics.com). Statistical tests between population means were first confirmed to have equal variance by *F* test, then analyzed using two-tailed Student's *t* test. Linear regressions were carried out using the least squares method. The LINEST Excel function was used for regression analysis of ribosomal gene twin-domains.

## RESULTS

### Measuring genome supercoiling in vivo by psoralen mapping

**Figure 1.**
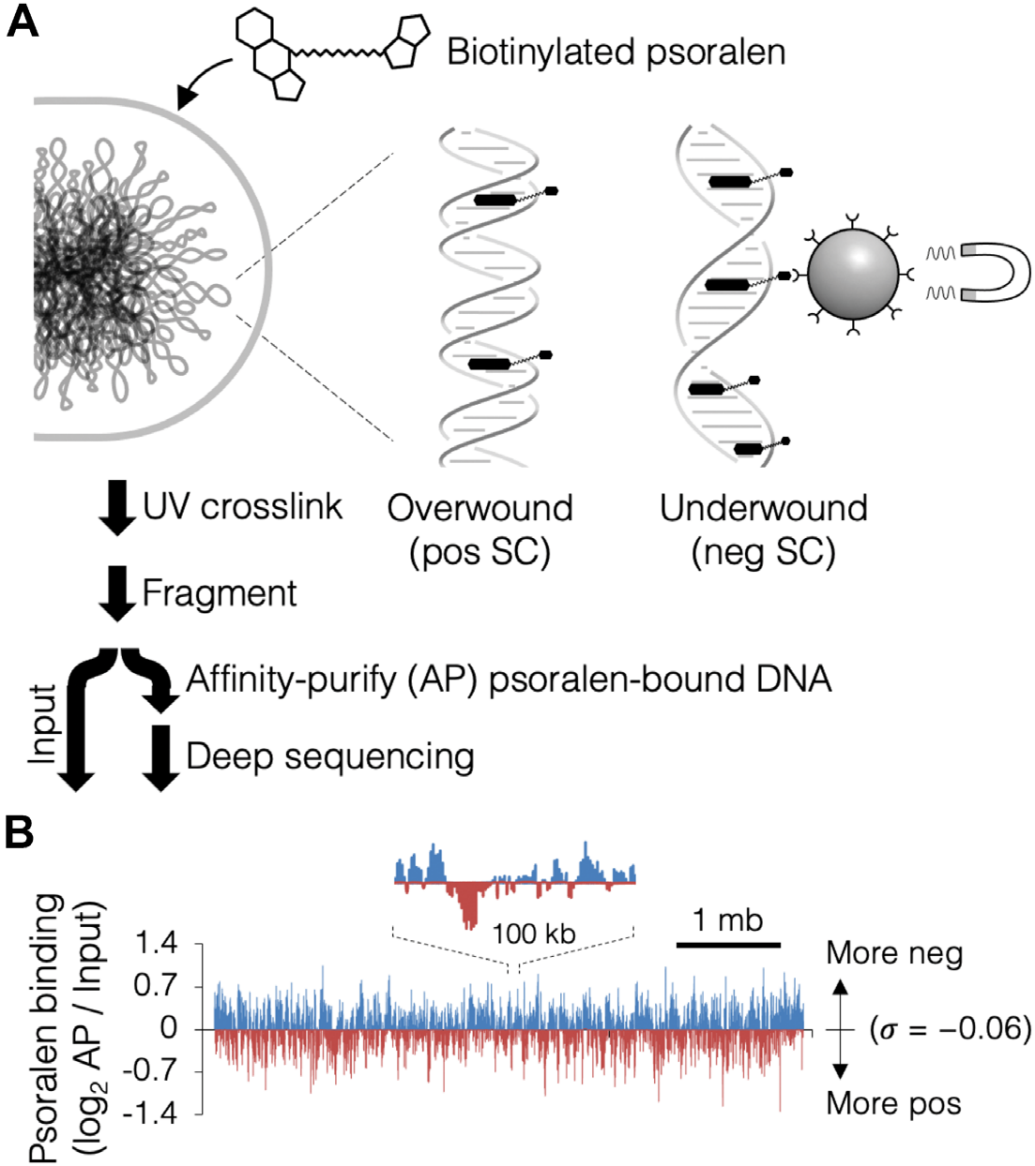
Psora-seq supercoiling assay. (**A**) Schematic of Psoralen crosslinking and DNA purification steps. Live cells are treated with biotinylated psoralen, which binds most strongly to highly negative supercoiled regions and most weakly to highly positive supercoiled regions. Psoralen–DNA adducts are crosslinked with UV light, genomic DNA is prepared and fragmented, then psoralen-bound fragments are affinity-purified using streptavidin magnetic beads. Pull-down and input samples are identified and quantified by whole genome sequencing. (**B**) Psora-seq profile of the *E. coli* genome. Binding is calculated as the number of sequencing reads per kb in the pull-down sample relative to the input sample, then log_2_ transformed. Final values indicate fold-difference in psoralen binding, with values above and below the genome average colored blue and red, respectively. Profile is from a single Psora-seq experiment with wild-type cells at mid-exponential phase in LB medium at 37°C.

Much of the difficulty typically experienced with mapping genomic supercoiling with psoralen stems from inefficient formation and reversal of UV crosslinks between psoralen molecules and bound DNA. Resulting low yields of psoralen-bound DNA result in a low signal to noise ratio and thus a loss of information on regions with less extreme supercoiling. To obtain reliable high-resolution supercoiling data, we developed a genomic psoralen binding assay that builds on previous methods but does not rely on onerous gel-excision (28) or microarray technology (23), which we termed Psora-seq (Figure 1A). Data collection is streamlined by utilizing a biotin-tagged version of psoralen, enabling pull-down with streptavidin, which greatly reduces non-specific interactions common with antibody-based immunoprecipitations. After brief incubation of cells with psoralen, crosslinking is catalyzed by high-intensity long-wave UV light, then genomic DNA is prepared and fragmented. Psoralen-bound fragments are affinity purified using magnetic streptavidin beads, then sequenced to a read depth of ∼50–100 (deep sequencing). A mock sample (Input) of the same cells without affinity purification is also sequenced, which serves as a reference for genomic copy number (origin-proximal DNA is more abundant) and sequencing bias. The ratio of the number of reads per kb in the affinity-purified sample to the number of reads per kb in the input sample is directly equivalent to the relative enrichment of psoralen binding along the chromosome. The ratios are then logarithmically transformed to indicate the fold-change in psoralen binding relative to the genome average. Finally, data from multiple independent experiments are averaged to further increase signal to noise ratio.

A typical Psora-seq profile from a culture of exponentially growing wild-type cells is shown in Figure 1B. Relative to average, psoralen-enriched regions (blue) are more negatively supercoiled and psoralen-depleted regions (red) are more positively supercoiled. Although the absolute values of supercoiling (σ) cannot be determined from this data since psoralen binding is a relative measurement, other experiments indicate that on average the genome is slightly under-twisted to σ = −0.06 (36), which corresponds to about six negative supercoils per kilobase. In our assay, psoralen binding varied by as much as 3-fold across the chromosome, about four times that reported in a previous *E. coli* study (28) when plotted with equal binning and smoothing. Most psoralen binding values are normally distributed about the mean of σ = −0.06, but there is a slight positive skew on a q-q plot (Supplementary Figure S1), suggesting that some loci exist in an extremely under-twisted state. Additionally, we did not observe a significant bias in psoralen binding for A + T rich DNA, indicating that G + C content corrections are unnecessary for the *E. coli* genome (Supplementary Figure S2).

### Supercoiling landscape of the *E. coli* chromosome

**Figure 2.**
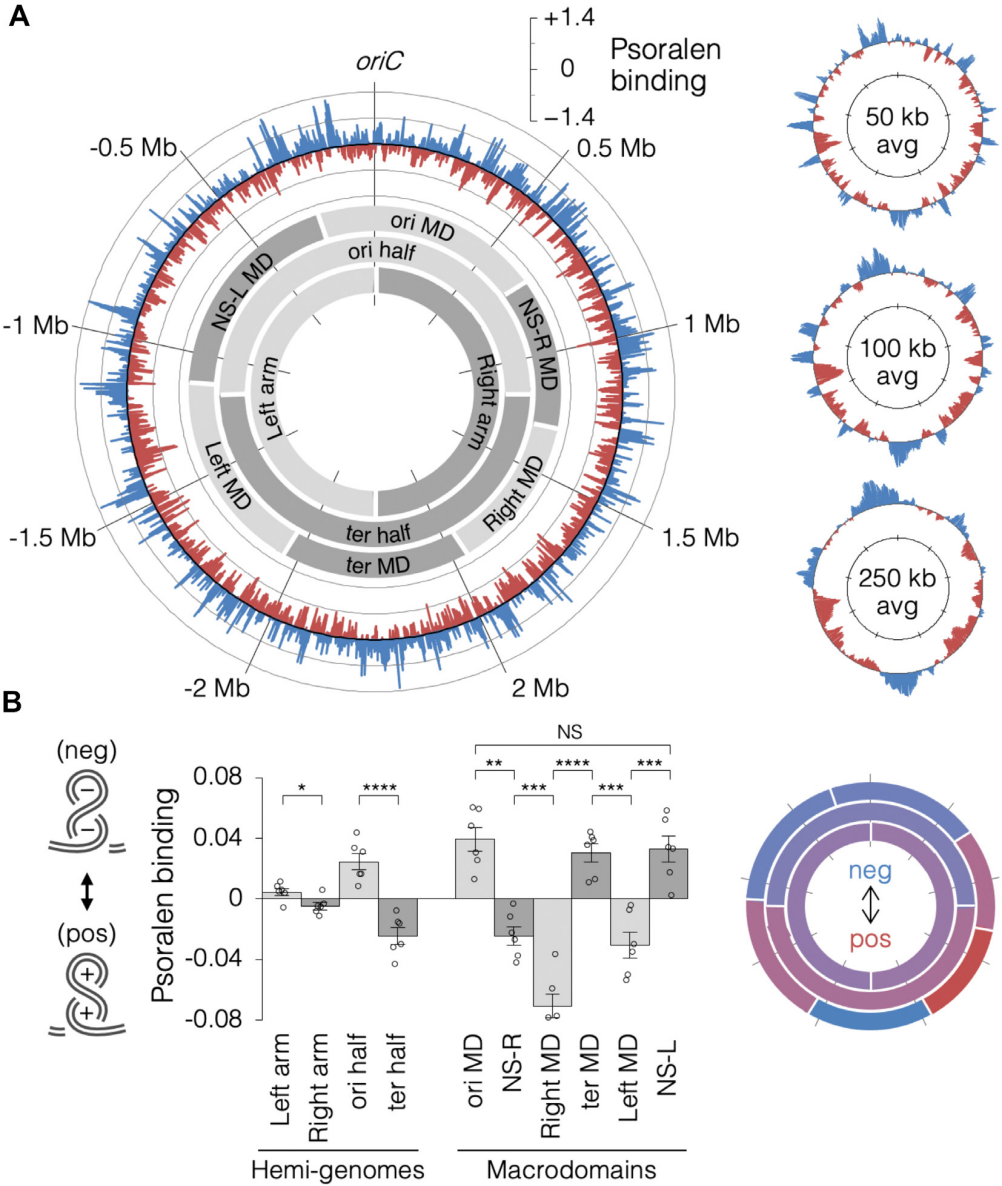
Large-scale supercoiling features of the *E. coli* chromosome. (**A**) Circular Psora-seq maps at 1-kb resolution (large plot on left) or at various moving averages (right plots). Psoralen binding values (log_2_ pull-down/input) are the average of six independent Psora-seq experiments with mid-exponential wild-type cells. Blue and red tracks indicate supercoiling that is more negative or more positive than the genome average, respectively. Higher-resolution extended plot shown in Supplementary Figure S3. Shaded grey arcs indicate the positions of macrodomains and hemi-genome regions. (**B**) Quantification of psoralen binding within hemi-genome and macrodomain regions (left) and corresponding heatmap (right). Error bars indicate ± s.e.m.; ****P< 0.0001; ***P < 0.001; **P < 0.01; *P < 0.05; NS = not significant (*p*> 0.05); two-tailed *t* test with df = 5 (*n* = 6 independent experiments).

Data from 6 independent Psora-seq experiments performed with wild-type cells at mid-exponential growth phase in rich medium were averaged and plotted on a circular chromosome map (Figure 2A). The resulting psoralen binding profile shows many large-scale variances ranging in size of 50–100 kb, with smaller-scale variances down to 5 kb visible on an extended plot (Supplementary Figure S3). Psora-seq profiles were highly reproducible, with an average correlation of *r* = 0.75 ± 0.09 (Supplementary Figure S4A). Large-scale supercoiling variances were dependent on psoralen (Supplementary Figure S4B) and were strongly diminished (−58 ± 6%) in cells exposed to bleomycin (Supplementary Figure S4C-E), which creates DNA single and double strand breaks, thereby relaxing DNA supercoiling (40). Analysis of hemi-genome regions defined by the replication origin and path of bidirectional replication forks showed that supercoiling was very similar on the left and right chromosome arms, while conversely, the top origin-containing half of the chromosome was significantly more negative than the terminus half of the chromosome (Figure 2B). We next quantified psoralen binding within the six macrodomain regions of *E. coli*, which have been shown to have elevated intra-contact frequency (33) and exhibit independent compaction and motion (32,49). Interestingly, psoralen binding differed significantly between adjacent macrodomains (Figure 2B). This finding is compatible with the idea that macrodomain boundaries and other chromosome interaction domains (CIDs) are topological barriers (33,50). Inspection of CID boundaries show them to be enriched in highly expressed genes (33,50), and it is possible that transcribing RNAP at these locations blocks diffusion of supercoils (51). However, our data did not indicate that structured macrodomains (ori, Left, Right and ter) are more negatively supercoiled than the two non-structured macrodomains (NS-L and NS-R), as has been predicted (32,33,49).

**Figure 3.**
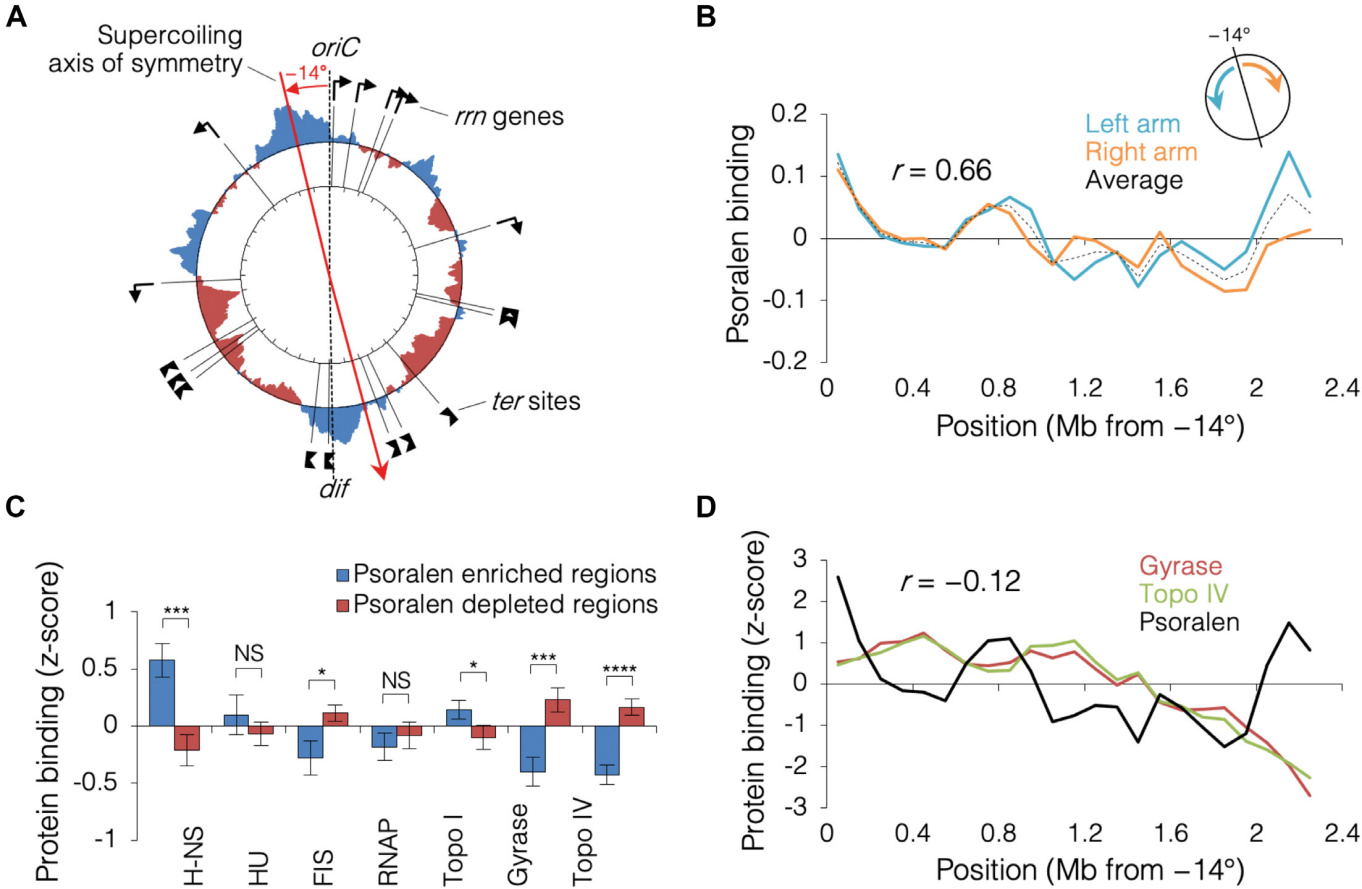
Symmetrical supercoiling about a skewed *ori*-*ter* axis. (**A**) Psoralen binding profile (250 kb moving average) from mid-exponential wild-type cells (*n* = 6; data from Figure 2) is shown with positions of ribosomal operons and Tus-binding termination (*ter*) sites. Blue and red tracks indicate supercoiling that is more negative or more positive than the genome average, respectively. (**B**) Psoralen binding (100 kb moving average) along left (cyan line) and right (orange line) chromosome arms displaced leftward by 14°. Average of left and right arms is shown (black dashed line). Determination of optimal skew shown in Supplementary Figure S5. (**C**) Binding of nucleoid proteins and topoisomerases within the most negative and most positive supercoiled regions. Genomic protein binding data for H-NS (27), HU (45), FIS (27), RNAP (27), Topo I (GEO GSM1696179), gyrase (46), and Topo IV (46) was standardized (*z*-score) and average binding was determined within psoralen enriched (blue) and psoralen depleted (red) regions (Materials and Methods). Error bars indicate ± s.e.m.; ****P < 0.0001; ***P < 0.001; **P < 0.01; *P < 0.05; NS = not significant (*p*> 0.05); two-tailed *t* test with df = 17 (*n* = 18 psoralen enriched regions, *n* = 24 psoralen depleted regions). Genome-wide protein correlation analyses are shown in Supplementary Figure S6. (**D**) Gyrase and Topo IV binding along skewed *ori-ter* replication axis. Protein binding *z*-scores as in (C) were averaged within 100 kb bins along left and right chromosome arms skewed leftward as in (B).

An additional large-scale feature visible in the Psora-seq data is an apparent 2-fold (mirror) symmetry between supercoiling of the left and right chromosome arms (Figure 3A). Symmetry was quantified by determining the Pearson correlation coefficient between the psoralen profiles of the left and right arms (Supplementary Figure S5A). Interestingly, symmetry was strongest (*r* = 0.66) when the axis of symmetry was skewed counterclockwise of the natural *ori*-*ter* axis by 14 degrees (Figure 3B; Supplementary Figure S5B-D). Although the causal mechanism of this symmetry is unknown, it seems likely that it is related to DNA replication. This is supported by prior evidence that leftward replication precedes rightward replication by 5–10 min (52,53) and genetic elements that inhibit replication, ribosomal genes and replication termini, are also symmetrical about a left skewed *ori-ter* axis (outer black symbols, Figure 3A). Thus, supercoiling is seemingly balanced along the paths of left and right replication forks, which we speculate might function to synchronize progression of sister replisomes (discussion).

We next analyzed binding data for major nucleoid proteins and topoisomerases against our Psora-seq data. This analysis showed that H-NS protein, which forms DNA-DNA bridges capable of constraining negative supercoils (54) and restricting short-range interactions with surrounding DNA (33), is preferentially bound to negatively supercoiled regions (Figure 3C; Supplementary Figure S6A). HU, FIS and RNAP binding were not significantly correlated to supercoiling (Figure 3C; Supplementary Figures S6B-D). Conversely, both type-II topoisomerases, gyrase and Topo IV, are preferentially bound to positively supercoiled regions (Figure 3C; Supplementary Figures S6E-F), and their binding is generally stronger near the origin and weaker near the terminus (Figure 3D). Additionally, both topoisomerases are more active, as determined by mapping cleavage complexes (46,48), within psoralen depleted (positively supercoiled) regions (Supplementary Figure S6H-J). This may reflect a strong recruitment of these topoisomerases to their target substrate, positively supercoiled DNA, and immediate dissociation after DNA relaxation (10,55). Supporting this interpretation, binding of Topo I, a type-I topoisomerase largely responsible for relaxing negative supercoils (22), has a slight preference for negatively supercoiled DNA (Figure 3C; Supplementary Figure S6G). Lastly, amidst the mostly positively supercoiled terminus half of the chromosome, we observed a ∼100 kb region of negative supercoiling centered at the *dif* locus within the terminus region (Figure 3A, D). This is unexpected given that convergence of replication forks in this region should create positive supercoils (3). A possible clue to this puzzle comes from Topo IV cleavage data, which shows a peak of very high activity at the *dif* locus (46; Supplementary Figure S7). Given that Topo IV has been shown to relax positive supercoils (56) in addition to its function as a decatenase (57), we speculate that the broad peak of negative supercoiling seen at the terminus may result from diffusion of negative supercoils generated by Topo IV at *dif*.

### Supercoiling twin-domains of ribosomal RNA genes

**Figure 4.**
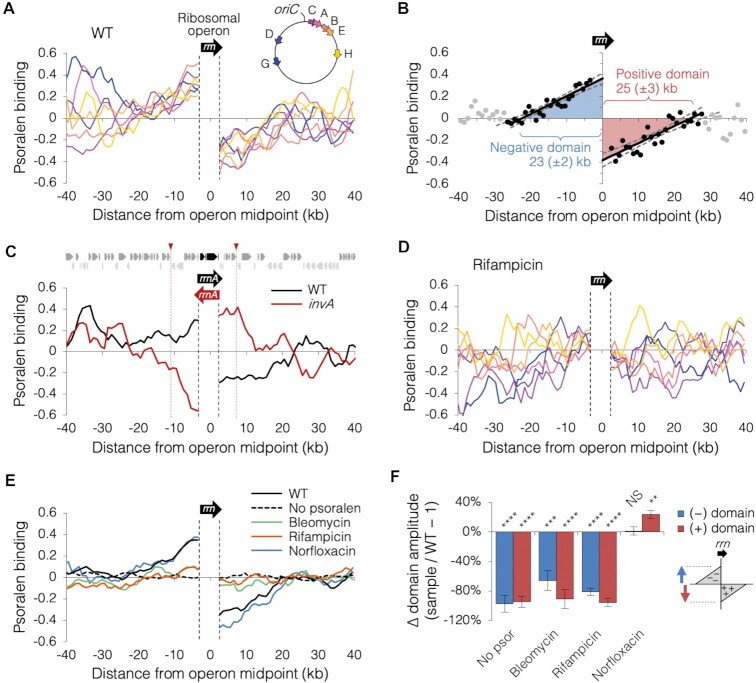
Twin-domain supercoiling at ribosomal operons. (**A**) Psoralen binding along an 80-kb region surrounding each of the seven ribosomal operons in mid-exponential wild-type cells (*n* = 6; data as in Figure 2). Psoralen profiles were co-oriented such that transcription is in the left to right direction. Sequencing reads within ribosomal gene open reading frames (dashed lines) is highly repetitive and thus cannot be mapped. (**B**) The consensus ribosomal operon twin-domain. Average psoralen binding near all 7 ribosomal operons in 6 independent experiments was plotted as in (A) and twin-domain size was determined by regression analysis. Linear regression lines (thick black lines) on each side of the operon were drawn using the set of contiguous data points (dark circles) that resulted in the maximum Pearson correlation coefficient. Length (magnitude) and height (amplitude) of each domain is given by the corresponding regression x- and y-intercepts. Statistical ranges were determined from the intercepts of 95% confidence intervals (dashed lines). (**C**) Inversion of the *rrnA* operon results in an oppositely oriented twin-domain. Average psoralen binding is shown for wild-type (black, *n* = 6) and *invA* mutant cells (red, *n* = 3) carrying an 18.4 kb inversion (red arrowheads) around the *rrnA* locus (39). Transcription profile and psoralen regression analysis is shown in Supplementary Figure S8. (**D**) Psoralen binding at ribosomal operons after rifampicin treatment. Mid-exponential wild-type cells were treated with rifampicin for one hour before crosslinking (*n* = 4). Profiles plotted as in (A). (**E**) Changes in ribosomal supercoiling after drug treatment. Average psoralen binding at seven aligned ribosomal operons is shown for: untreated cells (WT; *n* = 6; data as in Figure 2), cells put through a mock pull-down without psoralen (*n* = 2), cells treated with bleomycin for 10 minutes (*n* = 2), cells treated with rifampicin for one hour (*n* = 4), or cells treated with norfloxacin for 20 min (*n* = 2). Raw psoralen profiles shown in Supplementary Figure S9. (**F**) Change in supercoiling domain amplitude after drug treatment. Positive and negative supercoiling domain amplitudes were determined for above samples (E) by regression analysis as in (B). Error bars indicate ± s.d.; ****P < 0.0001; ***P < 0.001; **P < 0.01; *P < 0.05; NS = not significant (*p*> 0.05); two-tailed *t* test with df = 1–3.

As a test of Psora-seq's ability to measure changes in supercoiling occurring at the gene level, we analyzed psoralen binding around the 7 ribosomal gene operons, which together represent as much as 70% of transcriptional activity under rapid growth conditions (12). Although not yet directly observed on a chromosome in vivo, actively transcribing RNA polymerase complexes are thought to form two flanking (twin) domains of supercoiling, a positive supercoiling domain ahead (downstream) of the complex and a negative supercoiling domain behind (upstream) of the complex (7). Indeed, examination of Psora-seq data around each ribosomal operon indicates that a dramatic change in supercoiling occurred on both sides of the open reading frames (Figure 4A). Psoralen binding was highest and lowest immediately upstream and downstream of the operons, respectively, indicating maximal negative and positive supercoiling occurred at the flank of each transcription unit. Both positive and negative supercoiling domains declined gradually to baseline within 20–30 kb of the operon midpoint.

To determine the strength (amplitude) and range (magnitude) of twin-domains generated at ribosomal operons, psoralen binding at all 7 operons was averaged and twin-domain dimensions were analyzed by regression (Figure 4B). The resulting ‘consensus’ ribosomal twin-domain profile is an average of 7 operons × 6 independent samples and thus has exceptional resolution. Linear regression analysis on either side of the operon included the set of consecutive points (kb) that resulted in the maximum coefficient of correlation (black dots; Materials and Methods). Regression lines (solid black lines) crossed the x-axis at 23.1 ± 1.8 kb upstream (left) of the operon and 24.6 ± 2.7 kb downstream (right) of the operon, denoting the average length (magnitude) of negative and positive supercoiling domains, respectively. Domain strength (amplitude) was 0.38 ± 0.06 fold-change above and below the baseline, corresponding to a 38 ± 6% increase or decrease in psoralen binding from the local average. Thus, ribosomal gene transcription caused a significant supercoiling distortion that diffused throughout a 48 kb region.

To confirm that disrupted psoralen binding around the ribosomal genes is caused by supercoiling and not by direct exclusion by RNAP as has been suggested (58), we tested a strain carrying an 18 kb inversion including the *rrnA* ribosomal operon (39), resulting in an opposite orientation of *rrnA* transcription. As expected, the *rrnA* inversion strain (*invA*) exhibited a reversed twin-domain at the *rrnA* locus compared to wild-type (Figure 4C). Quantification of twin-domain size by regression analysis showed that the positive and negative domains in the inverted strain were similar in amplitude and magnitude to wild-type, but with an opposite orientation (Supplementary Figure S8). We further confirmed the dependence of twin-domains on transcription by performing Psora-seq on cells treated with rifampicin, which blocks RNAP elongation, for one hour before crosslinking. Resulting profiles showed an 88 ± 5% reduction in ribosomal gene twin-domain amplitude compared to untreated cells (Figure 4D–F), indicating that twin-domains were dependent on active transcription. Loss of twin-domain supercoiling from rifampicin treatment was similar to that seen after nicking the chromosome with bleomycin or in a mock pulldown without psoralen (Figure 4E, F; Supplementary Figure S9B, C). Treatment of cells with norfloxacin, which inhibits both type-II topoisomerases, gyrase and Topo IV, for 20 min before crosslinking resulted in a slight increase (24 ± 4%) in amplitude of the positive supercoiling domain (Figure 4E, F; Supplementary Figure S9D). This result supports a model in which gyrase removes positive supercoiling generated by transcription complexes (22). This rather modest effect of norfloxacin may result from the rapid blockage of transcription complexes in the absence of gyrase; supported by the finding that cell growth is stopped, and all transcription-dependent supercoiling is ended by 60 min of norfloxacin treatment (Supplementary Figure S9E, F).

**Figure 5.**
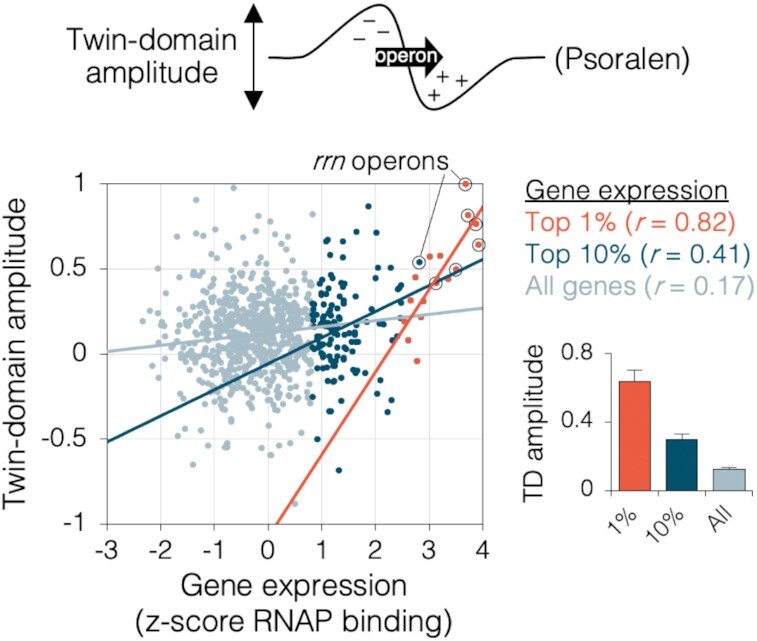
Transcription-dependent supercoiling for all genes. Twin-domain amplitudes for all 2,598 *E. coli* transcription units (837 multi-gene operons and 1761 single genes) were calculated as the difference in psoralen binding 5 kb downstream and upstream of each transcription unit (*n* = 6; data from Figure 2). Gene expression was calculated as the cumulative RNAP binding (43) within each transcription unit, converted to standard (*z*) score. Ribosomal operons are indicated with a circle. Inset graph shows average twin-domain amplitude among the top 1% or 10% expressing genes, or all genes (right). Error bars indicate ± s.e.m. (*n* = 6 independent experiments).

Based on this data, we would predict that all genes generate twin-domains, with dimensions analogous to those observed at ribosomal genes, but whose amplitudes scale to their relative rates of transcription. To test this idea, we examined psoralen binding around all 2598 transcription units (837 multi-gene operons and 1761 single genes) for which we obtained transcription data. Measuring twin-domain amplitude as the change in psoralen binding across the operon (Figure 5, top diagram), we observed a weak positive relationship (*r* = 0.17) between transcription strength (RNAP binding x operon length) and twin-domain amplitude among all genes (Figure 5, light blue), suggesting that many genes create twin-domains that are measurable above local supercoiling signal (background). This correlation was much higher among the most highly transcribed genes, with *r* values of 0.41 and 0.82 for the top 10% expressed genes (dark blue) and top 1% expressed genes (red), respectively. Because twin-domain amplitudes did not scale proportionally with transcription among weakly transcribed genes, we expect weaker twin-domain signatures are masked by nearby transcription or other modulators of supercoiling. Supporting this conclusion, ribosomal operon twin-domain amplitude was reduced by about 40% during late-exponential phase, which has ∼10-fold lower rate of rRNA transcription initiation compared to mid-exponential phase, and twin-domains were completely absent during stationary phase (Supplementary Figure S10).

### Transcription data as a predictor of global genome supercoiling

**Figure 6.**
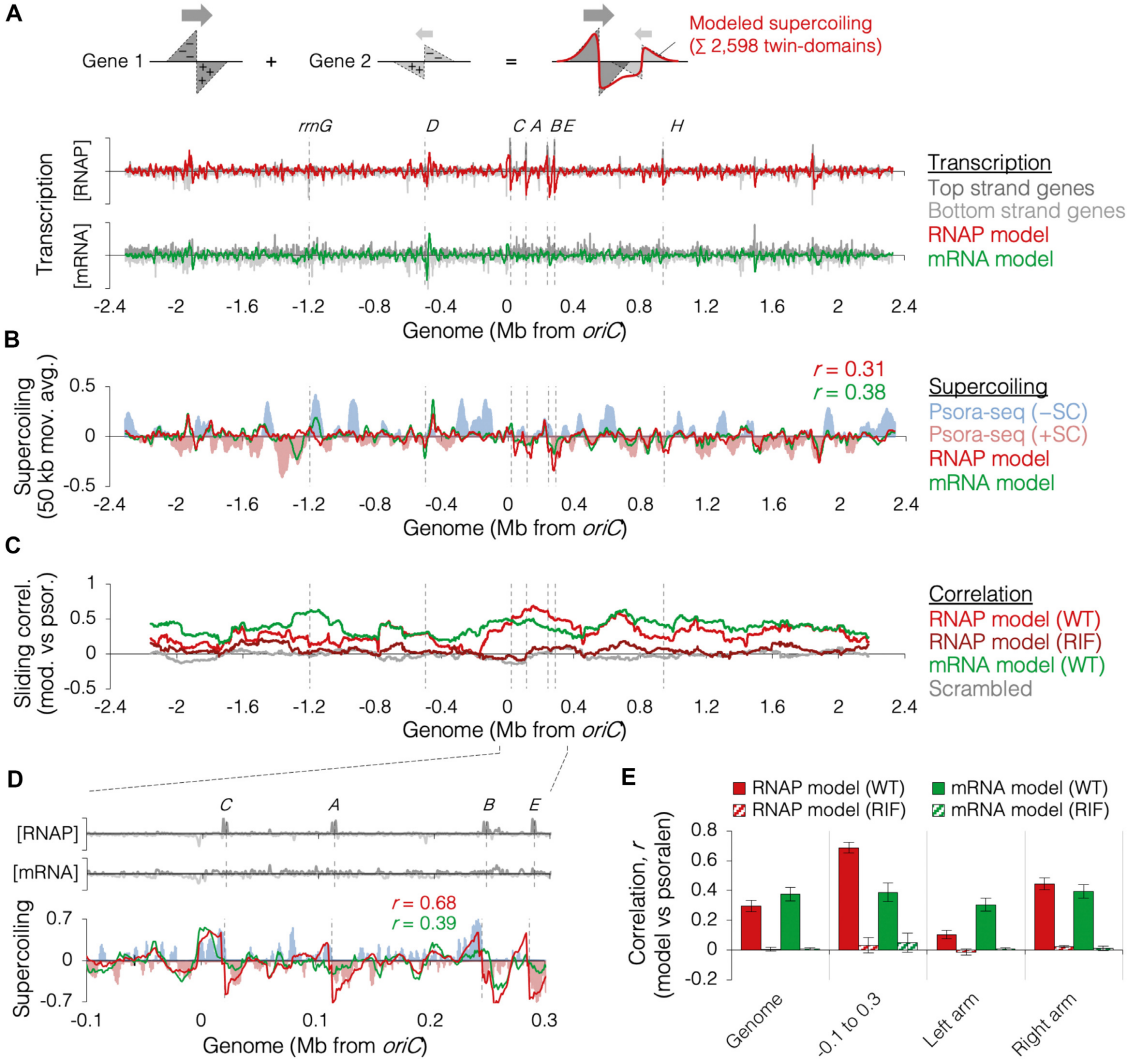
Modeling genomic supercoiling from transcription. (**A**) Modeling method and resulting modeled supercoiling profiles. Drawing illustrates how supercoiling is modeled at two converging genes. *E. coli* transcriptome (grey tracks) from RNAP binding (top; 43) or relative mRNA abundance (bottom; 59) is shown with modeled supercoiling (red and green lines) at 1-kb resolution. Dashed vertical lines mark the locations of ribosomal operons. (**B**) Genome supercoiling measured by Psora-seq (light blue and light red tracks) with RNAP and mRNA transcription models (red and green lines, respectively). Data are smoothed to a 50 kb moving average. (**C**) Sliding correlation plot showing model fitness along the chromosome. Pearson correlation coefficients were determined within a 300-kb window every 1 kb between Psora-seq data from mid-exponential wild-type cells (*n* = 6; Figure 2) and RNAP-modeled supercoiling (red) or mRNA-modeled supercoiling (green), between scrambled Psora-seq data and RNAP-modeled supercoiling (grey), or between Psora-seq data from rifampicin-treated cells and RNAP-modeled supercoiling (dark red). Detailed modeling analysis of rifampicin-treated cells is shown in Supplementary Figure S11. (**D**) Zoomed plot of a region with high transcription. RNAP (*rrn* depleted) and mRNA transcriptomes (top) and supercoiling profiles from Psora-seq or transcription models (bottom) are shown as above (A, B). (**E**) Model fitness within different chromosome regions. Error bars indicate ± s.e.m. (*n* = 6 Psora-seq experiments).

Because the twin-domain supercoiling signatures of many genes is discernable in the psoralen binding data, we hypothesized that the combined twin-domain effects of all genes might accurately predict global genomic supercoiling. To understand the impact of multiple transcription units and their additive effects on neighboring supercoiling, individual supercoiling twin-domains were modeled from transcriptome data, then summed across the genome (drawing, Figure 6A). Twin-domains were modeled with constant magnitudes of 23 kb for the negative domain and 25 kb for the positive domain as determined for ribosomal genes (Figure 4B). Amplitudes of individual operons were scaled to transcriptome data from two different studies, quantifying transcription from RNAP binding (43) or mRNA abundance (59). RNAP binding data has the advantage of including ribosomal genes, while mRNA data was performed with the same strain and under the same growth conditions as the current study. Summation of all 2958 twin-domains and scaling to Psora-seq data rendered two models of genomic supercoiling based solely on transcription (Figure 6A, dark red and green lines). Both models showed moderate agreement with Psora-seq data (Figure 6B).

To determine which regions of the chromosome are modeled well and which are modeled poorly, we determined the correlation between modeled supercoiling and Psora-seq data in a 300-kb sliding window across the chromosome. This analysis shows that both models predicted supercoiling reasonably well throughout the chromosome (Figure 6C, red and green lines) with genome-wide correlations of *r* = 0.31 ± 0.05 for the RNAP data and *r* = 0.38 ± 0.04 for the mRNA data (Figure 6E). By comparison, a scrambled psoralen data set (grey line) showed no relation to either transcription model (*r* = 0.002 and *r* = 0.005). Treatment of cell cultures with rifampicin one hour before psoralen crosslinking resulted in correlations of *r* ≤ 0.01 to both transcription models (Figure 6C, E; and Supplementary Figure S11), a reduction of 98 ± 1% from untreated cells. Furthermore, genome-wide psoralen binding in rifampicin treated cells was about half of untreated cells (47 ± 5%, Supplementary Figure S12), indicating that much of the psoralen signal observed was transcription-dependent.

Interestingly, model fitness was generally higher at the replication origin and over the first ∼1/3 of the right chromosome arm (Figure 6C, E). As many highly transcribed genes reside in this region (47), we speculate that model correlation improves with increased gene expression. This is supported by an expanded view of a region near *oriC* including four ribosomal operons (Figure 6D) in which supercoiling is very well correlated to the RNAP binding model (*r* = 0.67, red line) but relatively modestly correlated to the mRNA model (*r* = 0.39, green line), which does not include expression data for ribosomal genes. High correlation between RNAP model and Psora-seq data was present at all 7 ribosomal operons (*r* = 0.71 ± 0.05, Supplementary Figure S13). Importantly however, reasonable correlation between supercoiling and the mRNA model, which excludes all *rrn* genes, indicates that the relationship between transcription data and DNA supercoiling is not dependent on ribosomal genes.

## DISCUSSION

The helical nature of DNA presents a topological problem for DNA translocases such as DNA and RNA polymerases, which act as moving topological barriers. The twin-domain model of Liu and Wang (7) predicts that rotation of polymerase complexes is inhibited by connections to the cell membrane and/or drag within the cytoplasm. Consequently, torsional strain is introduced proportionally to the deficit between the rates of polymerase rotation and helix unwinding (60). Analysis of DNA supercoiling by Psora-seq at the seven *E. coli* ribosomal operons showed that these transcriptional units produced nearly symmetrical ‘twin’ negative and positive supercoiling domains each about 25 kb in length (Figure 4B). Both domains exhibited maxima at the gene border with linear decay kinetics. To our knowledge, this is the first in vivo demonstration of a chromosomal transcription twin-domain with both overwound and underwound supercoiling domains in any system. Although ours is the first psoralen-based assay of ribosomal gene supercoiling in *E. coli*, previous estimates of the maximum distance of topological influence from transcription are significantly less than what we measured. For example, in vivo measurements of the distance of diffusion of transcriptional-dependent supercoiling, either from inter-gene activation/repression assays (13,14,29) or mapping of a positive-supercoiling dependent binding protein (58), are limited to about 2–8 kb, equal to one-third to one-tenth the distance measured by Psora-seq.

Genomic psoralen-based studies indicate that eukaryotic transcription-dependent supercoiling is significantly weaker and less diffusive than in bacteria, often with no positive supercoiling detected at all (23,38,41,61). Several factors may contribute to this effect, including a different consortium of topoisomerases in eukaryotes, such as the RNAP-associated (21) type IB topoisomerase (topoisomerase I), which can relax both negative and positive supercoils (62) unlike bacterial Topo I, which can only relax negative supercoils (18). Additionally, ribosomal gene transcription initiation rates in exponentially growing *E. coli* (∼60 init/min/gene) exceed the highest expressed genes in eukaryotes by a factor of ∼2–4 (12,63). Supporting this conclusion, we found that ribosomal gene twin-domain height (amplitude) was about 40% lower during late-exponential phase, presumably a consequence of decreased transcription rates (12).

We found the genomic supercoiling landscape revealed by Psora-seq could be reasonably predicted from transcription data alone (Figure 6), suggesting that transcription is the primary determinant of total genomic supercoiling. A central role of transcription in genome supercoiling should not be unexpected given that individual twin-domains extended over 20 kb in each direction, and the *E. coli* genome is very gene-dense with an average inter-operon distance of less than 1 kb. Additionally, our data is consistent with a wide store of molecular and genetic data showing that impairment of transcription via rifampicin or *rpoB* mutation causes a rapid topological change to bacterial chromosomes including: a two-fold expansion of the nucleoid (64), suppression and enhancement of topoisomerase mutations (65), and a genome-wide change in γδ recombination (6). The ubiquitous signature of transcriptional twin-domains in Psora-seq data also indicates that topological changes imposed by transcription are near the buffering capacity of topoisomerases. This contrasts with the view that topoisomerases are in excess and remove polymerase-generated supercoils as they are formed (19,66). Like transcription-mediated supercoiling, there is also compelling evidence that extensive supercoiling is generated during DNA replication. However, unlike RNAP, which only synthesizes over very short stretches, DNA polymerase remains bound for many hundreds or thousands of kilobases, potentially generating immense supercoiling fields on either side of the replication fork. Torsional stress at replication forks routinely builds to the point that sister chromatids become entwined, creating long catenated structures (9,52,67). These temporary entanglements are important for homologous recombination and chromosome segregation (9,67). Thus, changes in supercoiling imposed by transcription and replication complexes are long-lived and instrumental for chromosome function.

Psora-seq measurements have also provided insight into domain structure of the genome. Early work in *E. coli*, quantifying the number of γ-radiation induced double strand breaks to fully relax chromosomes by psoralen binding (30) or testing for a change in transcription of supercoiling-sensitive promoters after cleavage of intact genomic DNA with a restriction endonuclease (29), suggest that domain barriers have an average spacing of about 10 kb (29) to 100 kb (30). A less invasive method in *Salmonella* Typhimurium testing for recombination between pairs of γδ transposons placed around the chromosome, which only occurs if they reside in the same topological domain, suggests that barriers have an average spacing of about 25 kb (31). Additionally, there is compelling evidence that topological barriers are randomly placed from cell to cell (29,31). Our observation that twin-domain supercoiling displays smooth and linear decay from both sides of the ribosomal operons (Figure 4B), also suggests that supercoil diffusion is blocked by randomly positioned topological barriers. The average distance between barriers is given by the maximal distance of supercoil diffusion, about 24 kb, equating to about 200 topological barriers per genome. We note however, that this interpretation assumes that supercoiling diffusion is virtually instantaneous, and that topoisomerase activity is evenly distributed around ribosomal genes. It may also be possible that extremely high transcription at ribosomal genes creates supercoiling ‘tidal waves’ that sweep across one or more topological barriers, resulting in an underestimation of the number of topological domains from twin-domain data.

Our data also supports the view that *E. coli* macrodomains are mediated by DNA supercoiling. These 600–800 kb domains have been shown to have distinct compaction and mobility characteristics (68). Although we did not find that the four structured macrodomains had higher supercoiling levels than the two non-structured macrodomains, as postulated (32), we did find that adjacent macrodomains have significantly different supercoiling (Figure 2). This supports recent 3C experiments showing that transitions (nodes) between regions of high interaction, presumably governed by DNA topology, are located at or near macrodomain boundaries, suggesting that macrodomain boundaries are also topological barriers (33). Inconsistence between our data and an earlier report showing that macrodomains had similar supercoiling levels (6) may be due to their testing only a single site in each macrodomain.

Psoralen mapping also revealed two interesting features of genome supercoiling related to DNA replication. First, while average supercoiling of the left and right chromosome halves was nearly identical, supercoiling of the upper and lower halves differed significantly, with the upper (origin) half being more negative and the lower (terminus) half being more positive (Figure 2). Supercoiling was not homogenously negative or positive within each hemi-genome, but instead was characterized by several broad peaks or groups of peaks along both halves, suggesting that the difference between the *ori* and *ter* halves is due to discrete genetic elements along the chromosome. Analysis of genomic binding data for the major nucleoid proteins showed that H-NS had a moderate preference for negatively supercoiled DNA (Figure 3C; Supplementary Figure S6), possibly attributable to H-NS’s function as a transcription regulator (27). In contrast, we found almost no correlation between supercoiling and binding of FIS, which has been shown to reduce extreme supercoiling generated during exponential growth (69), or HU, which can stimulate negative supercoiling in the presence of Topo I (24,25). The later finding is supported by a similar result in a previous psoralen study (28) concluding that HU may play a larger role in governing supercoiling during stationary phase. Together, our data suggest that observed variances in supercoiling along the *E. coli* genome are mostly caused by the cumulative effects of transcription, rather than binding patterns of nucleoid associated proteins or topoisomerases. This conclusion is illustrated by the finding that gyrase and Topo IV are bound preferentially to positively supercoiled regions – the target substrate of these enzymes, not their product. The trend of more negatively supercoiled regions near the origin and more positively supercoiled regions near the terminus lends some support to a model in which gene expression is regulated by a gradient of supercoiling along the chromosome, with genes activated by negative supercoiling present more often near the origin and vice versa (17,70). However, our data suggest that global supercoiling patterns are driven by transcription rather than orchestrated gyrase and nucleoid protein binding as hypothesized. We speculate that elevated negative supercoiling observed near the origin reflects this region's higher gene density and favored relaxation of positive supercoiling over negative supercoiling at transcriptional twin-domains.

Supercoiling also showed remarkably high co-variation between left and right chromosome arms, or replichores (Figure 3). As the replichores define the paths of the left and right replication forks, which are often coordinated in a single replication complex (replication factory;^71^), we speculate that symmetrical supercoiling results from coordinated progression of the two forks. Coupled progression of the two presumably large fluxes in supercoiling associated with the left and right replication forks (9) might facilitate a synchronized change in transcription along the replichores (11). Interestingly, supercoiling symmetry was highest along a skewed axis, rotated 14 degrees counterclockwise (Figure 3; Supplementary Figure S5). We speculate that the observed supercoiling pattern is a consequence of a delay in timing of the right replication fork relative to the left fork, which has been previously observed (52,53). Ironically, the timing disparity between left and right replication forks might be caused by transcription-mediated supercoiling effects from the two origin-flanking genes, *gidA* and *mioC* (59). The skew in replication timing and supercoiling symmetry may also explain the longstanding puzzle of why ribosomal genes and replication termination sites, likely the two most potent regulators of replication fork progression, are also more symmetrical about a 14-degree axis than the canonical *ori-ter* axis (Figure 3A). Future studies that include accurate measurements of genomic DNA supercoiling are needed to better understand the dynamic relationship between transcription and replication.

## DATA AVAILABILITY

All Psora-seq data generated in this study are available on the NCBI Sequence Read Archive (BioProject PRJNA805241). Custom Matlab scripts that were used to compile and sort sequencing data are available on the GitHub repository (https://github.com/DavidBatesLab/Matlab-scripts.git).

## Supplementary Material

gkac244_Supplemental_File
