# Supplementary material for: Psoralen mapping reveals a bacterial genome supercoiling landscape dominated by transcription
Source: Nucleic Acids Res. 2022 Apr 14;50(8):4436–49. doi: 10.1093/nar/gkac244 (PMC9071471; doi:10.1093/nar/gkac244)
Supplement: gkac244_Supplemental_File [file gkac244_supplemental_file.pdf]

SUPPLEMENTARY FIGURES

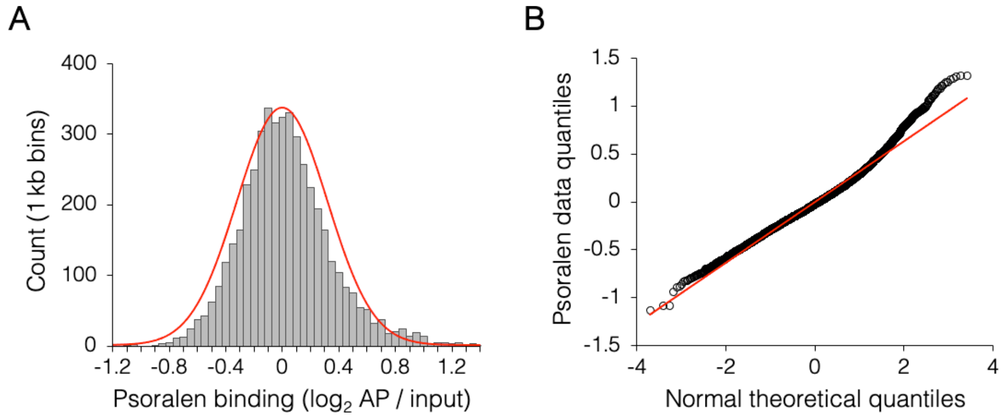

Supplementary Figure S1. Distribution of psoralen binding. (**A**) Frequency distribution of psoralen binding across the *E. coli* chromosome (grey bars), and normal curve (red). Psoralen data as in Figure 2 (n = 6). (**B**) Quantile-quantile (Q-Q) plot between psoralen binding data and an expected normal distribution. Deviation of right tail of psoralen data above normal curve indicates that psoralen data is positively skewed (more negatively supercoiled) from a normal distribution. (mean = 0; *s.d.* = 0.321; *s.e.m.* = 0.005).

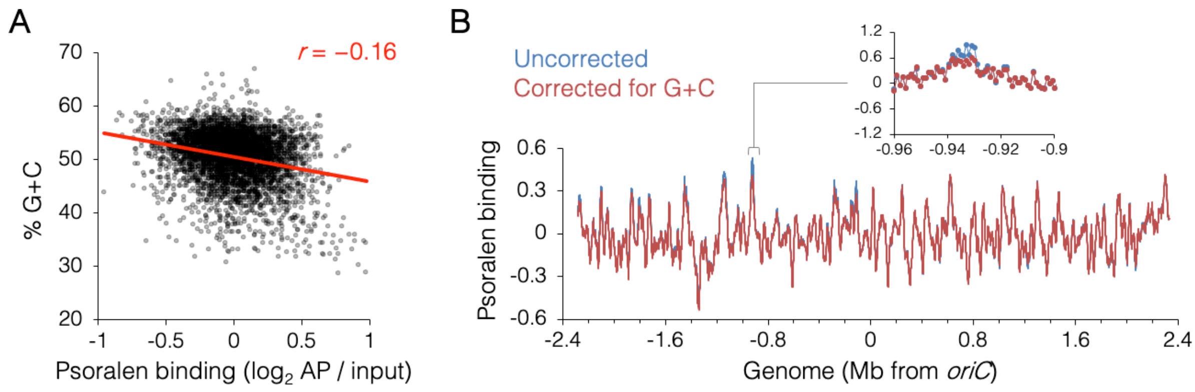

Supplementary Figure S2. Minimal effect of G+C bias on psoralen binding distribution. (**A**) Plot of psoralen binding and G+C content in 1-kb bins for the *E. coli* chromosome. Linear regression shown with Pearson correlation coefficient. Psoralen data as in Figure 2 (n = 6). (**B**) 10-kb moving average plot of psoralen binding profile across the genome before (blue) and after (red) correction for G+C content (G+C corrected = original psoralen binding in bin x [% G+C in bin / mean % G+C all bins]). Inset graph shows the region containing the maximal difference of G+C correction on the chromosome (1-kb resolution without smoothing).

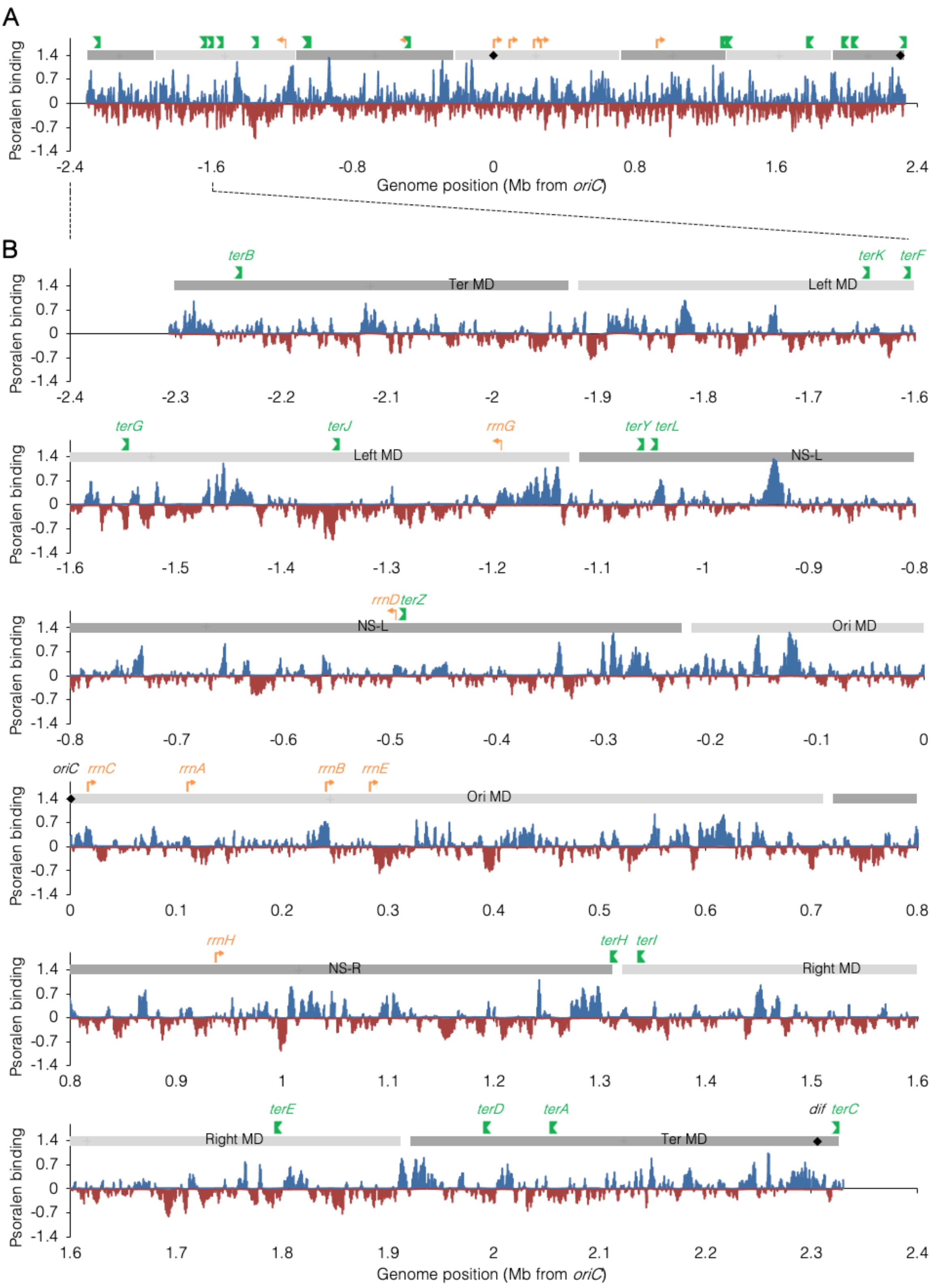

Supplementary Figure S3. High-resolution psoralen binding profile for the *E. coli* chromosome. (**A**) Condensed psoralen binding profile. Mean psoralen binding from mid-exponential wild-type cells is shown at 1-kb resolution (n = 6; data as in Figure 2). Blue and red tracks indicate supercoiling that is more negative or more positive than the genome average, respectively. Positions of macrodomains (grey bars), replication termini (green brackets), ribosomal operons (orange arrows), and *oriC* and *dif* loci (black diamonds) are indicated. (**B**) Zoomed psoralen binding profile as in (A).

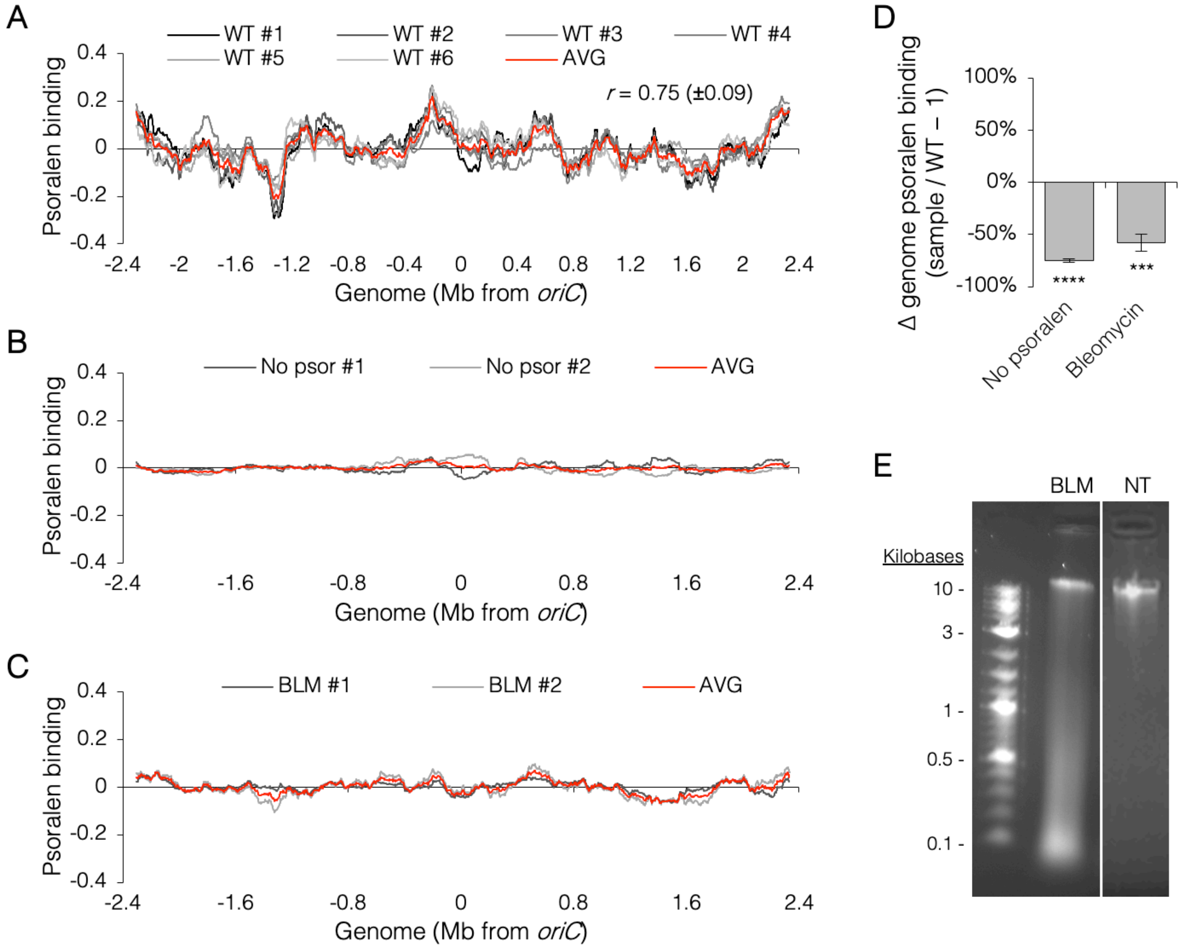

Supplementary Figure S4. Reproducibility of Psora-seq and negative controls. (**A**) Psoralen binding profiles for mid-exponential wild-type cells (*n* = 6; data as in Figure 2). Lines are 50 kb moving averages of single experiments (grey) and average (red). Pearson correlation (*r*) is the average of 15 correlations between all pairwise combinations of Psora-seq data (± s.d.). (**B**) Psora-seq profiles from mock pull-down experiments omitting psoralen (*n* = 2). Lines drawn as in (A). (**C**) Psora-Seq profiles from cells treated with the DNA nicking antibiotic bleomycin for 10 minutes prior to crosslinking (*n* = 2). Lines drawn as in (A). (**D**) Reduced genome-wide psoralen binding signal in no-psoralen and bleomycin treated samples. Fractions calculated as the sum of absolute psoralen binding values [(sample / WT) – 1]. (**E**) Chromosome fragmentation after 10 minutes bleomycin treatment. Genomic DNA from cells treated with bleomycin (C, BLM #1) and untreated cells (NT) was analyzed by electrophoresis in a 0.8% agarose gel prior to sequencing. DNA ladder is shown in left lane. Error bars indicate ± s.d.; ****$p<0.0001$; ***$p < 0.001$; **$p < 0.01$; *$p < 0.05$; NS = not significant ($p > 0.05$); two-tailed *t* test with df = 1.

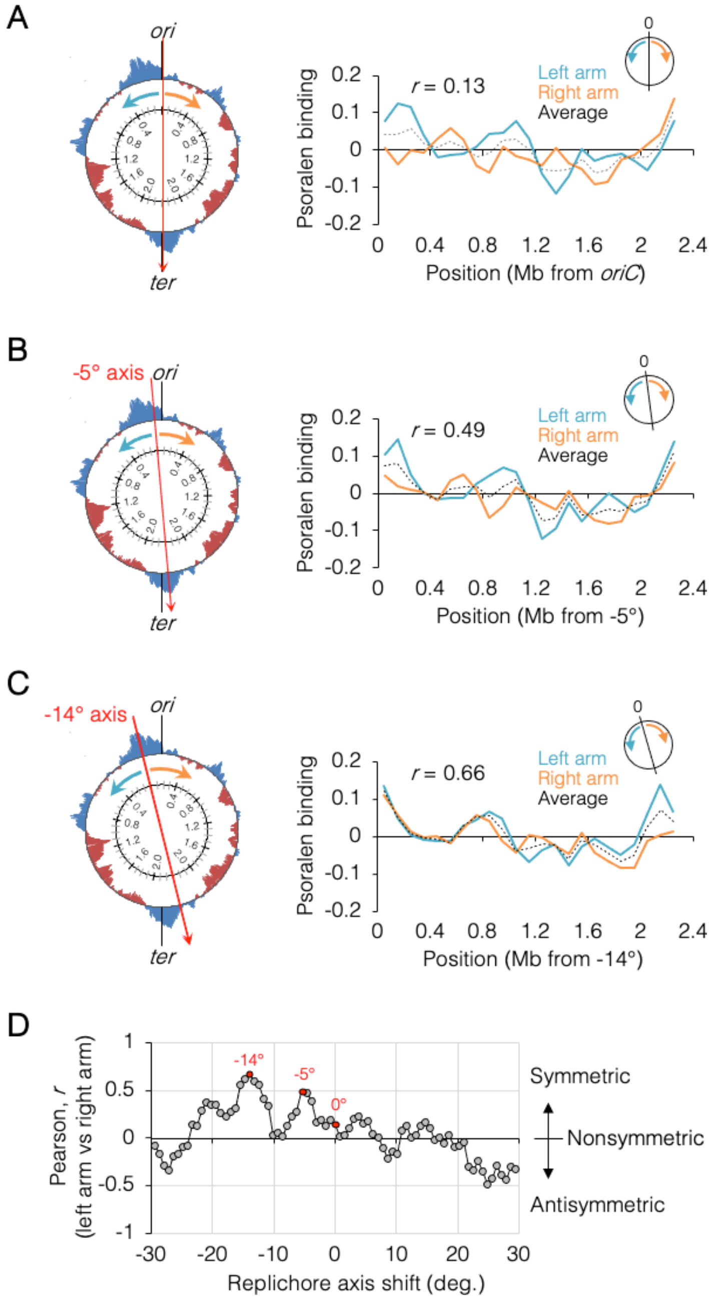

Supplementary Figure S5. Analysis of genome supercoiling symmetry. (**A**) Supercoiling symmetry with axis positioned at the natural *ori*-*ter* replication axis. Left: Circular supercoiling map showing 250 kb moving average of psoralen binding in mid-exponential wild-type cells (n = 6; data as in Figure 2). Tracks indicate regions that are more negative (blue) or more positive (red) than genome average. Right: Psoralen binding (100 kb bins) along left (cyan) and right (orange) chromosome arms, and average of left and right arms (dashed line). Pearson correlation between left and right arms is shown. (**B**) As in (A) but *ori*-*ter* axis skewed by -5 degrees. (**C**) As in (A) but *ori*-*ter* axis skewed by -14 degrees. (**D**) Correlation between left and right arm supercoiling for *ori*-*ter* axis skews of -30 to +30 degrees.

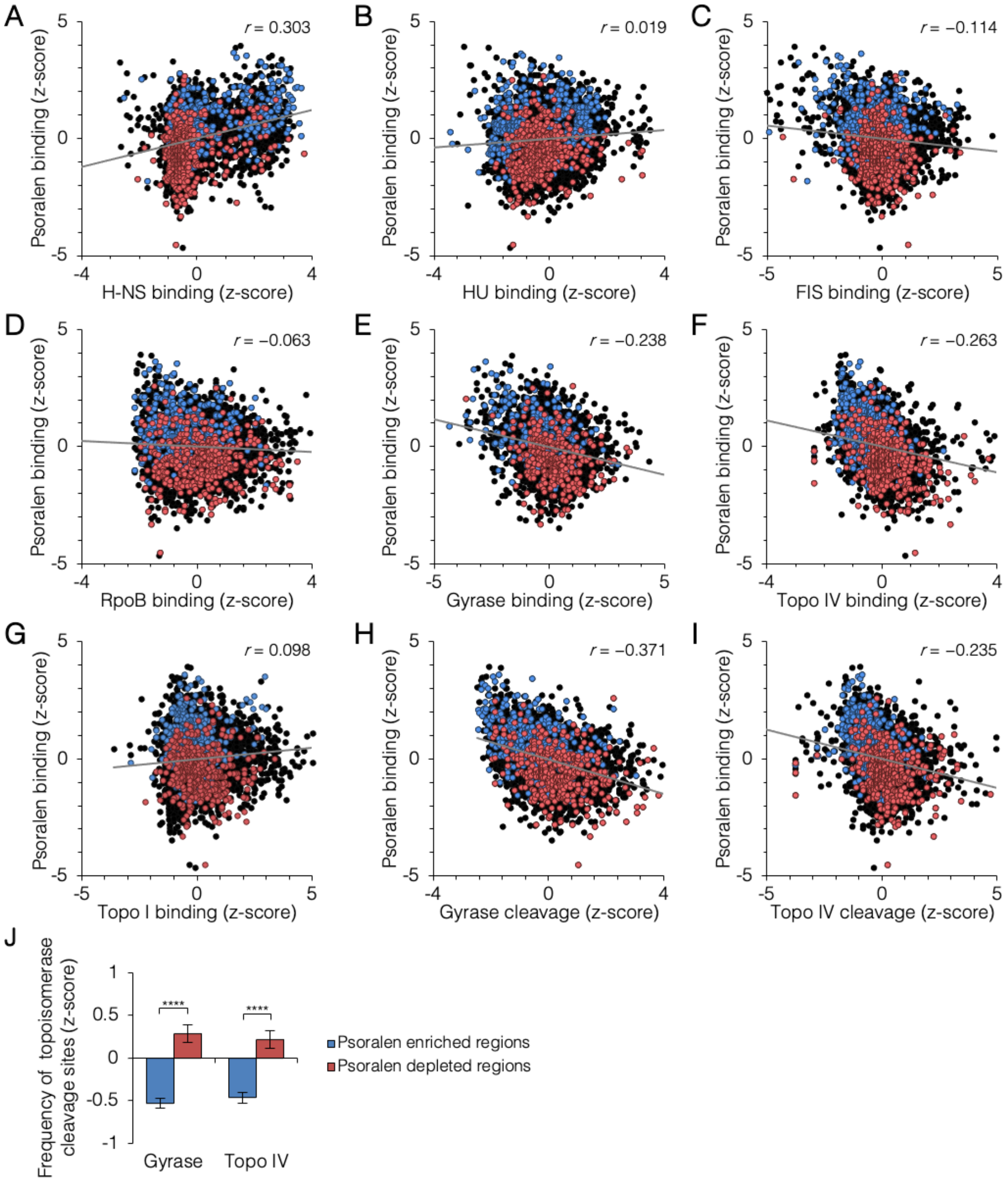

Supplementary Figure S6. Correlation analysis for binding of psoralen and supercoiling regulatory proteins. (**A-G)** Correlation scatterplots for binding of psoralen and 6 different regulatory proteins in 1-kb segments spanning the *E. coli* chromosome. Psoralen data is from mid-exponential wild-type cells (n = 6; data as in Figure 2). Genome binding information for nucleoid proteins and topoisomerases is ChIP-chip or ChIP-seq data from the following sources: H-NS (1), HU (2), FIS (1), RNAP (1), gyrase (3), Topo IV (3), and Topo I (GEO GSM1696179). All psoralen and protein binding data were apportioned into 1-kb bins, $\log_2$ transformed, then converted to standard (z) scores to facilitate comparisons (Materials and Methods). Blue symbols indicate psoralen-enriched regions and red symbols indicate psoralen-depleted regions as described in Figure 3. Pearson coefficients of correlation for the entire genome are shown. (**H-I**) Genomic correlation scatterplots for binding of psoralen and cleavage sites for Topo IV and gyrase. Frequency of double strand DNA cleavage complexes along the genome for Topo IV (3) and gyrase (4) was binned and converted to z-scores as in (A-G). (**J**) Frequency of Topo IV and gyrase cleavage within the most negatively supercoiled regions (psoralen enriched; blue) and within the most positively supercoiled regions (psoralen depleted; red). Error bars indicate ± s.d.; ****p<0.0001; ***p < 0.001; **p < 0.01; *p < 0.05; NS = not significant (p > 0.05); two-tailed t test with df = 1.

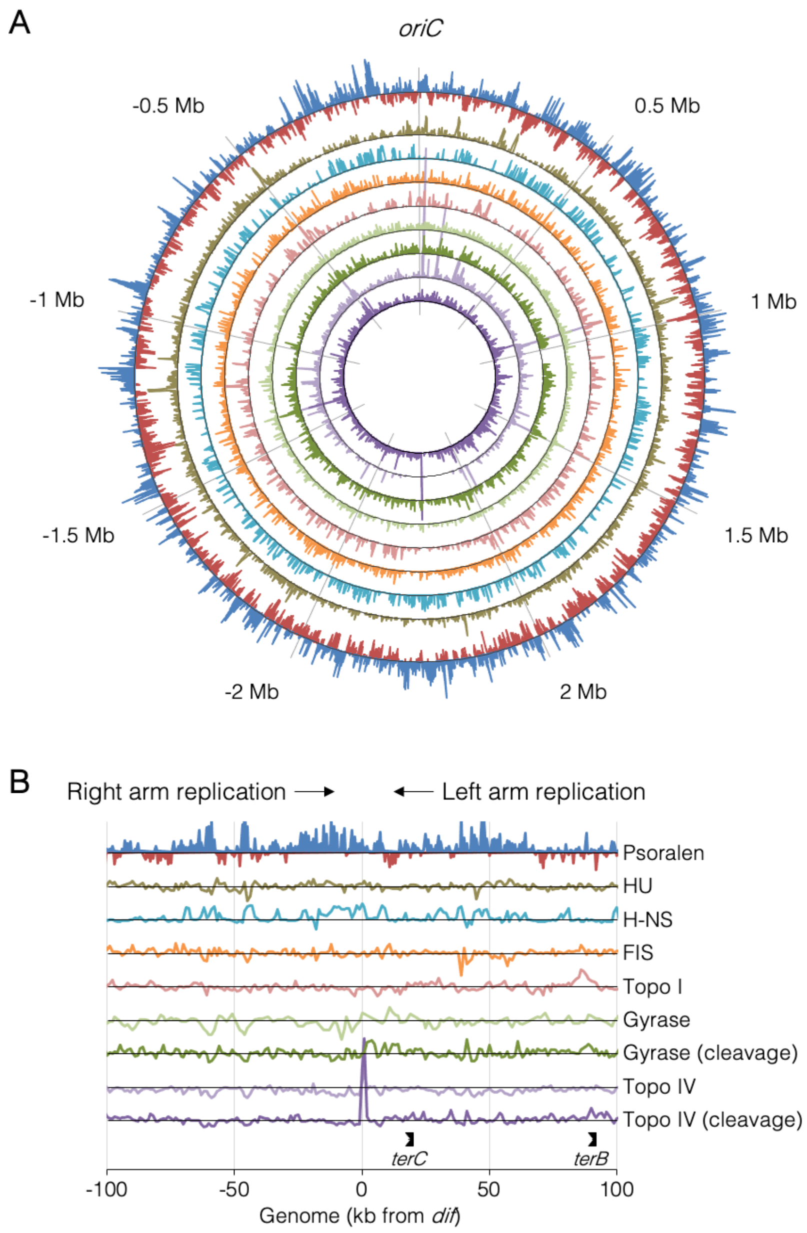

Supplementary Figure S7. Supercoiling and protein binding maps of *E. coli* genome and terminus. (**A**) Circular genome map including, from outer to inner ring: psoralen binding, HU binding, H-NS binding, FIS binding, Topo I binding, gyrase binding, gyrase cleavage, Topo IV binding, and Topo IV cleavage. Psoralen binding tracks indicate regions that are more negatively supercoiled (blue) or more positively supercoiled (red) than genome average. Source and handling of protein binding and cleavage data are described in Supplementary Figure S6. Psoralen binding data as in Figure 2 (n = 6). Lines are drawn at 1-kb resolution. (**B**) Zoomed terminus plot as in (A) showing prominent peak of Topo IV cleavage at the *dif* locus. Termination sites for rightward replication forks, *terB* and *terC*, are shown.

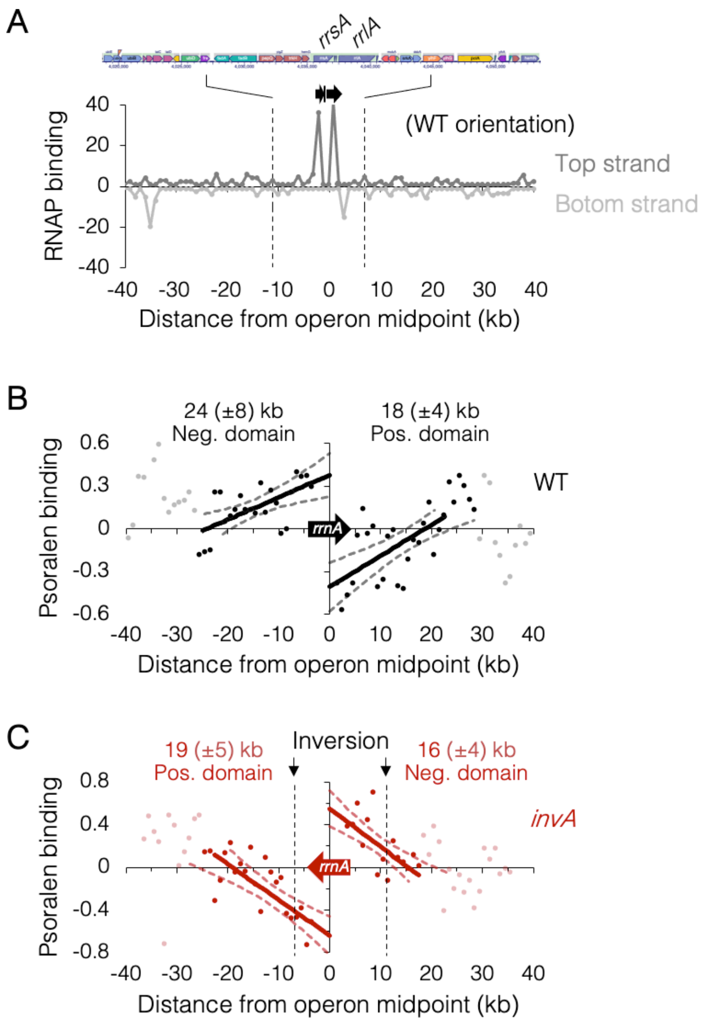

Supplementary Figure S8. Twin-domain analysis at an inverted ribosomal operon. (**A**) Genes and transcription profile around the *rrnA* operon. Genetic map (top graphic) and transcription levels from RNAP binding data (5) are shown for rightward (top strand) encoded genes and leftward (bottom strand) encoded genes. The positions of the *rrnA* operon (thick black arrows) and *invA* (6) inversion borders (dashed vertical lines) are indicated. (**B**) Psoralen binding for wild-type cells showing twin-domain with negative supercoiling upstream (left) of the operon and positive supercoiling downstream (right) of the operon (n = 6; data as in Figure 2). Regression and 95% confidence intervals were calculated as described in Figure 3B. Supercoiling domain magnitudes are indicated. (**C**) Psoralen binding and regression analysis for *invA* cells showing an oppositely oriented twin-domain (mean of n = 3 independent experiments). Because the inversion is not perfectly centered around the *rrnA* operon, there is a 6-kb shift in the sequences shown in (C).

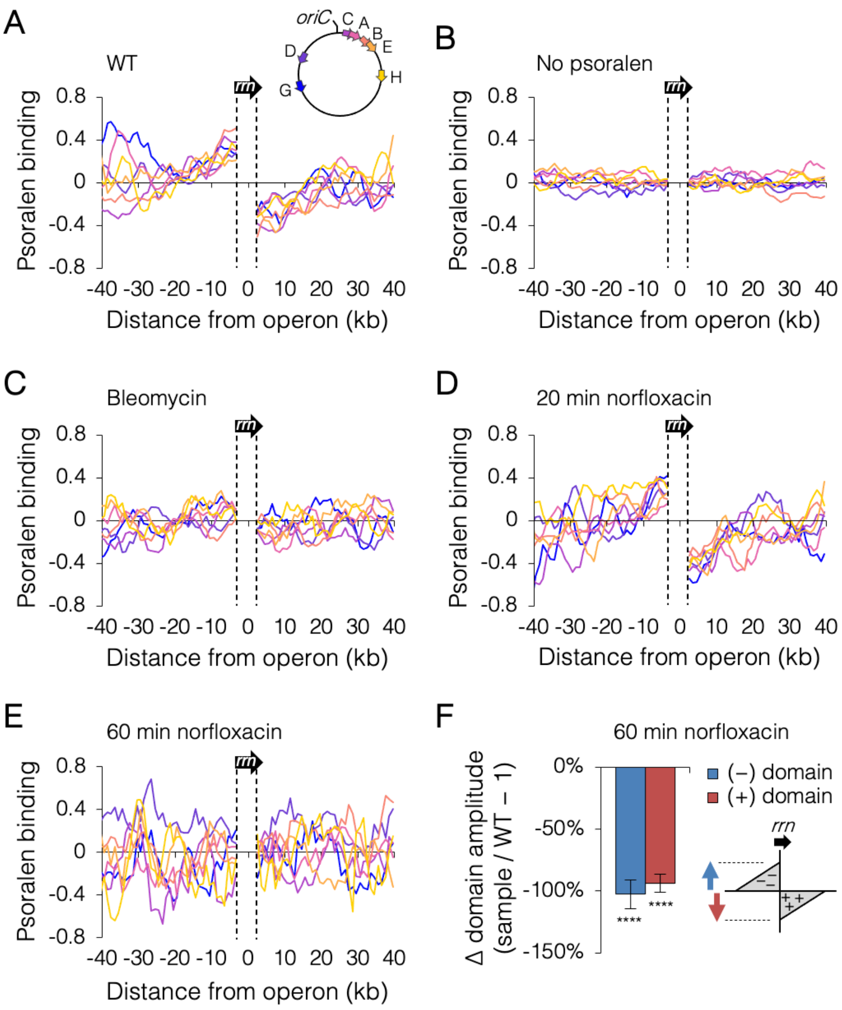

Supplementary Figure S9. Psoralen binding profiles at ribosomal operons after drug treatment. (**A**) Psoralen binding shown at all seven ribosomal operons in mid-exponential wild-type cells (*n* = 6) as described in Figure 4A. (**B**) Ribosomal operon psoralen binding profiles as in (A) for mock pull-down samples omitting psoralen (*n* = 2). (**C**) Ribosomal operon psoralen binding profiles as in (A) for cells treated with bleomycin for 10 minutes before crosslinking (*n* = 2). (**D**) Ribosomal operon psoralen binding profiles as in (A) for cells treated with nofloxacin for 20 minutes before crosslinking (*n* = 2). (**E**) Ribosomal operon psoralen binding profiles as in (A) for cells treated with nofloxacin for one hour before crosslinking (*n* = 2). (**F**) Change in supercoiling domain amplitude after 60 minutes norfloxacin-treatment, suggesting complete inhibition of transcription. Amplitudes were estimated by regression analysis as described in Figure 4.

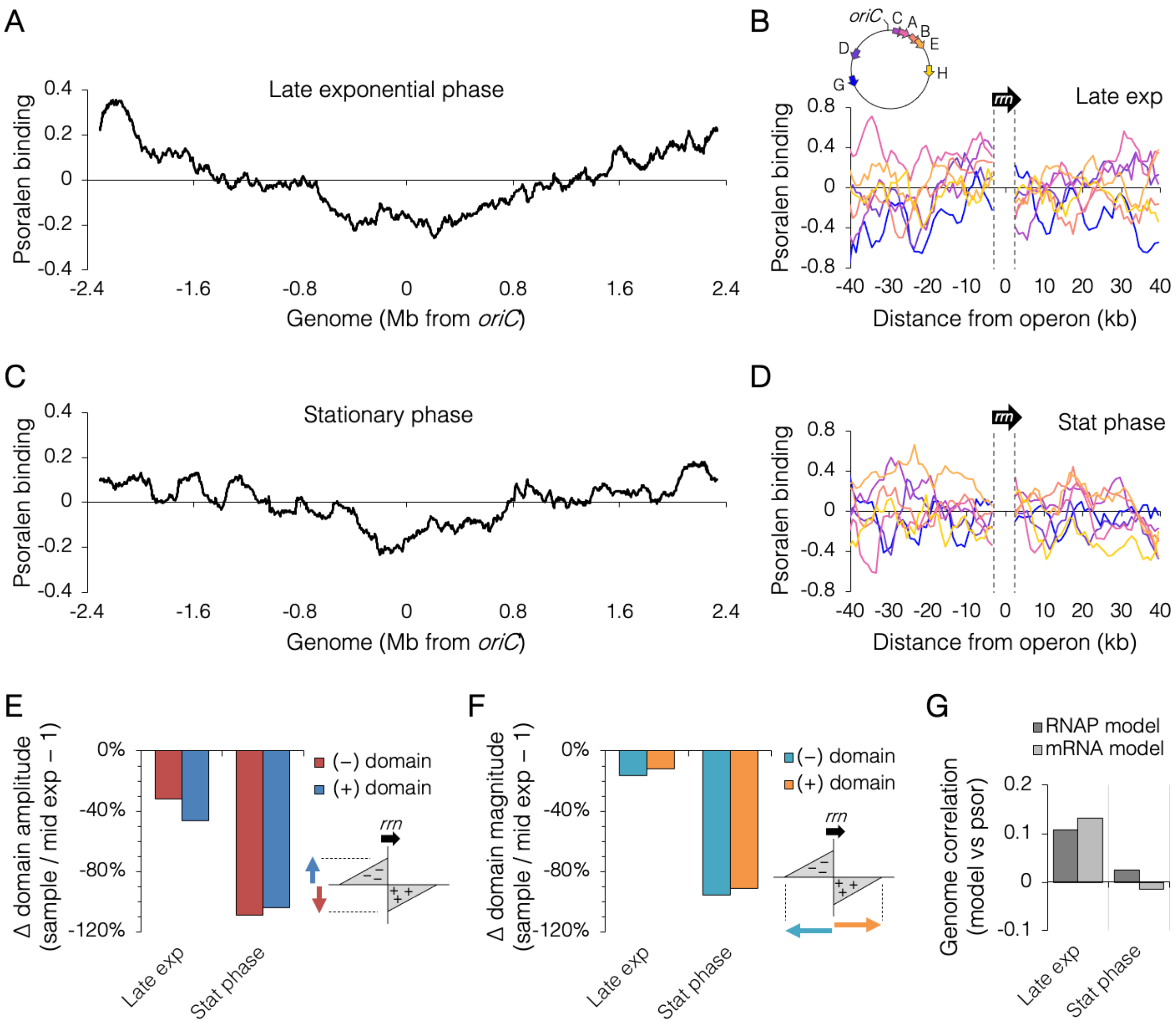

Supplementary Figure S10. Reduced ribosomal operon twin-domains during late-exponential phase and stationary phase. (**A**) Genome supercoiling profile for wild-type cells during late-exponential phase (O.D.600 = 2). Line is 50-kb moving average of psoralen binding (n = 1). (**B**) Psoralen binding shown at all seven ribosomal operons in cells shown in (A). (**C**) Genome supercoiling profile for wild-type cells during stationary phase (O.D.600 = 7). Line is 50-kb moving average of psoralen binding (n = 1). (**D**) Psoralen binding shown at all seven ribosomal operons in cells shown in (B). (**E**) Change in supercoiling domain amplitude in late-exponential and stationary phase cells shown in (A-D). (**F**) Change in supercoiling domain magnitude in late-exponential and stationary phase cells shown in (A-D). Amplitudes and magnitudes were estimated by regression analysis as described in Figure 4. (**G**) Genome-wide Pearson correlation between transcription models from exponentially growing cells and psoralen binding from late-exponential and stationary phase cells.

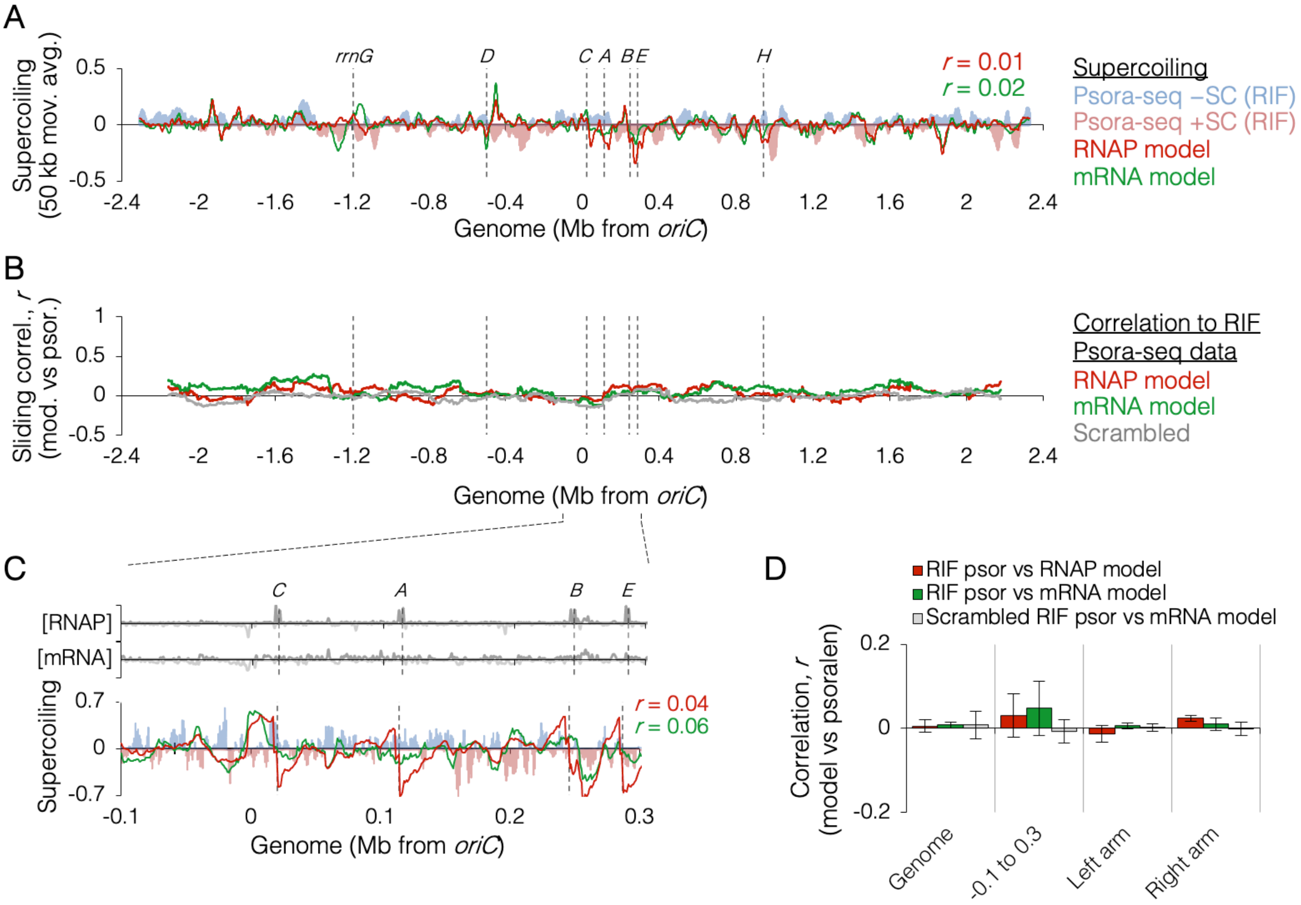

Supplementary Figure S11. Genomic supercoiling is uncorrelated to transcription after rifampicin treatment. (**A**) Supercoiling profile for rifampicin-treated cells and transcription models. Mid-exponential cells were treated with rifampicin for one hour before crosslinking (n = 4). Blue and red tracks indicate supercoiling (50-kb moving average of psoralen binding) that is more negative or more positive than genome average, respectively. Red and green lines indicate two models of supercoiling from transcription data, RNAP binding (red; 5) or mRNA abundance (green, 7). (**B**) Sliding correlation plot showing model fitness along the chromosome. Pearson correlation coefficients were determined within a 300-kb window every 1 kb between psoralen data from rifampicin-treated cells and supercoiling modelled from RNAP (red) or mRNA (green), or between scrambled psoralen data and RNAP model (grey). (**C**) Zoomed plot of a region normally having high transcription. RNAP and mRNA transcriptomes from wild-type cells (top) and supercoiling profiles from rifampicin-treated psoralen data or transcription models (bottom) are shown as in (A). (**D**) Model fitness within different chromosome regions. Error bars indicate ± s.e.m.

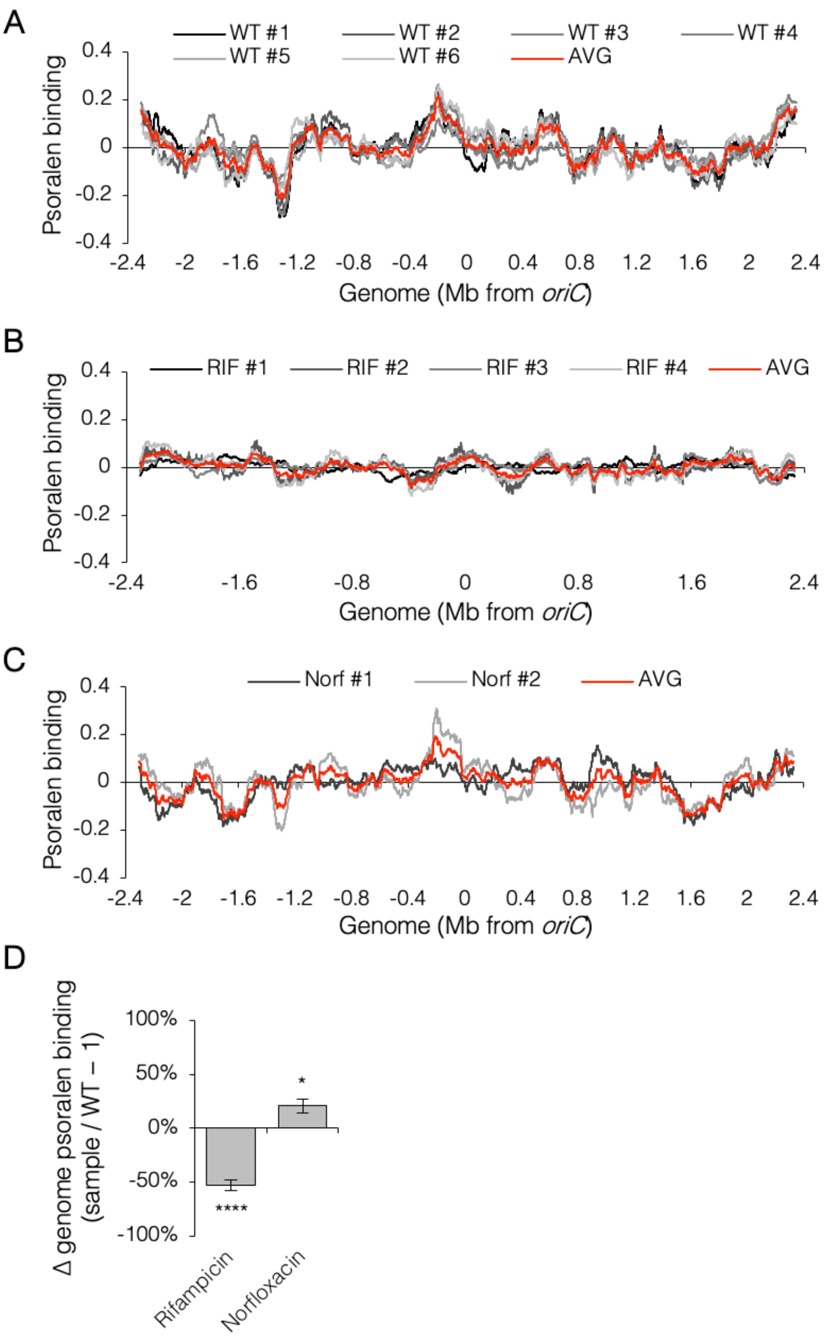

Supplementary Figure S12. Rifampicin treatment results in a flattened supercoiling profile. (**A**) Genomic psoralen binding profiles for untreated mid-exponential wild-type cells (n = 6; data as in Figure 2). Lines are 50 kb moving averages from single experiments (grey) and average (red). (**B**) Genomic psoralen profiles for mid-exponential wild-type cells treated with rifampicin for one hour before crosslinking (n = 4). Lines drawn as in (A). (**C**) Genomic psoralen profiles for cells treated with norfloxacin for 20 minutes prior to crosslinking (n = 2). Lines drawn as in (A). (**D**) Reduced genome-wide psoralen binding in rifampicin-treated cells. Fractions calculated as the sum of absolute psoralen binding values [(sample / WT) – 1]. Error bars indicate ± s.d.; ****p<0.0001; ***p < 0.001; **p < 0.01; *p < 0.05; NS = not significant (p > 0.05); two-tailed t test with df = 1.

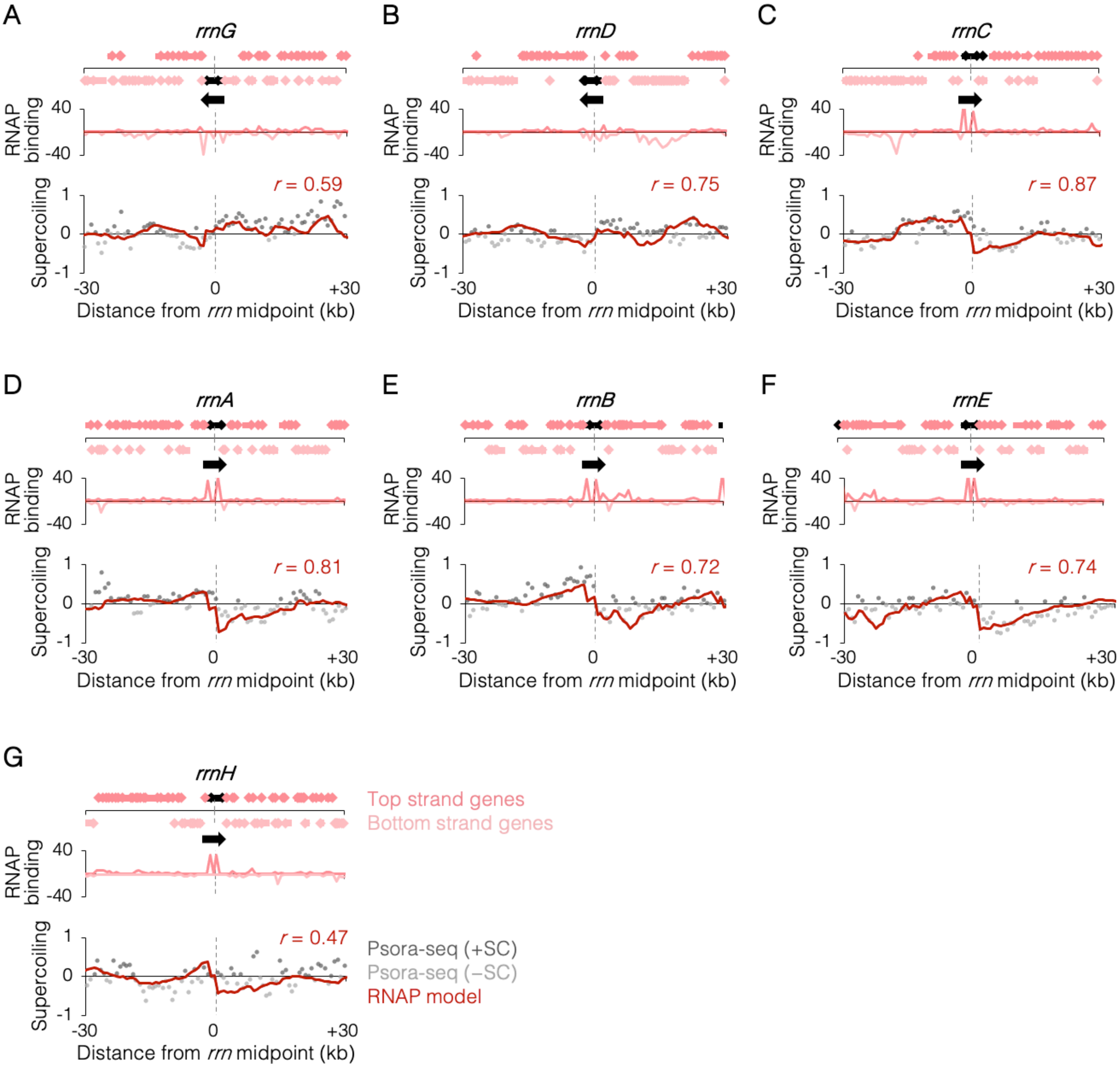

Supplementary Figure S13. Fitness of RNAP transcription model to psoralen profile at ribosomal operons. (**A-G**) Map of each ribosomal operon with transcriptome, transcription model, and Psora-seq data. Top: genes are shown for top strand (dark pink arrows) and bottom strand (light pink arrows) and ribosomal genes (black arrows). Middle: gene expression levels as relative RNAP binding (5). Bottom: psoralen binding (grey circles; *n* = 6; data as in Figure 2) and RNAP transcription model (dark red line). Pearson correlation coefficient between transcription model line and Psora-seq data are shown.

SUPPLEMENTARY REFERENCES

1. Kahramanoglou, C., Seshasayee, A.S., Prieto, A.I., Ibberson, D., Schmidt, S., Zimmermann, J., Benes, V., Fraser, G.M. and Luscombe, N.M. (2011) Direct and indirect effects of H-NS and Fis on global gene expression control in *Escherichia coli*. *Nucleic Acids Res*, **39**, 2073-2091.
2. Prieto, A.I., Kahramanoglou, C., Ali, R.M., Fraser, G.M., Seshasayee, A.S. and Luscombe, N.M. (2012) Genomic analysis of DNA binding and gene regulation by homologous nucleoid-associated proteins IHF and HU in *Escherichia coli* K12. *Nucleic Acids Res*, **40**, 3524-3537.
3. El Sayyed, H., Le Chat, L., Lebailly, E., Vickridge, E., Pages, C., Cornet, F., Cosentino Lagomarsino, M. and Espeli, O. (2016) Mapping topoisomerase IV binding and activity sites on the *E. coli* genome. *PLoS Genet*, **12**, e1006025.
4. Sutormin, D., Rubanova, N., Logacheva, M., Ghilarov, D. and Severinov, K. (2019) Single-nucleotide-resolution mapping of DNA gyrase cleavage sites across the *Escherichia coli* genome. *Nucleic Acids Res*, **47**, 1373-1388.
5. Grainger, D.C., Hurd, D., Goldberg, M.D. and Busby, S.J.W. (2006) Association of nucleoid proteins with coding and non-coding segments of the *Escherichia coli* genome. *Nucleic Acids Res*, **34**, 4642-4652.
6. Boubakri, H., de Septenville, A.L., Viguera, E. and Michel, B. (2010) The helicases DinG, Rep and UvrD cooperate to promote replication across transcription units in vivo. *EMBO J*, **29**, 145-157.
7. Lies, M., Visser, B.J., Joshi, M.C., Magnan, D. and Bates, D. (2015) MioC and GidA proteins promote cell division in *E. coli*. *Front Microbiol*, **6**, e00516.
